# Glioblastoma Cells Expressing Oncogenic EGFR Release Multiple Extracellular Vesicle Subpopulations Positive or Negative for EGFR

**DOI:** 10.1002/jex2.70134

**Published:** 2026-04-07

**Authors:** Elham Pishavar, Fereshteh Shojaei‐Ghahrizjani, Brian Meehan, Nadim Tawil, Cristiana Spinelli, Dongsic Choi, Fang Cheng Wong, Mahsa Jalali, Janusz Rak

**Affiliations:** ^1^ Research Institute of the McGill University Health Centre, Glen Site McGill University Montreal Quebec Canada; ^2^ Department of Biochemistry Soonchunhyang University College of Medicine Cheonan Republic of Korea

**Keywords:** cancer, EGFR, exosomes, extracellular vesicles, extracellular vesicle heterogeneity, glioblastoma, glioma, glioma stem cells, oncogene

## Abstract

Extracellular vesicle (EV) heterogeneity is well documented but poorly defined. This is especially important in cancer where EVs serve as carriers of unique oncogenic macromolecules that can be transferred to recipient cells or targeted for liquid biopsy diagnostics. Here we employed a series of human glioma cell lines to test the content and distribution of oncogenic epidermal growth factor receptor (EGFR) including its mutant (EGFRvIII) among different subsets of tumour‐derived EVs. Our results suggest that the global levels of EGFR packaged into EVs parallels its expression in parental cancer cells. However, while in glioma cells expressing high levels of EGFR (GSC83) this receptor was uniformly distributed on cellular surfaces, only a small fraction of their derived EVs contained EGFR, as documented by nano‐flow cytometry, ExoView and super‐resolution microscopy. Using only three protein markers (CD63, CD81 and EGFR) these single EV platforms revealed the existence of seven different EV subsets, of which four contained EGFR. Purified EGFR‐positive and negative EVs contained both shared and distinctive protein markers. EGFR packaging into EVs of GSC83 cells was independent of syntenin 1 expression, but was suppressed upon treatment with pharmacological inhibitor of neutral sphingomyelinase (GW4869). Exposure of human microglial cells (HMC3) to EVs released from GW4869‐treated and control glioma cells triggered distinctive changes in cellular proteome including transfer of EGFR. Overall, our results suggest that multiple pathways of EV biogenesis may operate in glioma cells resulting in formation of complex EV landscapes consisting of EGFR‐positive and EGFR‐negative EV subsets. This heterogeneity may have important implications for EV functions and EV‐based diagnostics.

## 1 Introduction

Transmembrane receptor tyrosine kinases (RTKs) are amongst the most studied oncogenic drivers and therapeutic targets in major human cancers, including, breast, lung and brain tumours (Ferguson and Gray 2018). Paradigmatic in this regard are the properties of the epidermal growth factor receptor (EGFR) and the related protooncogenes (HER2, 3, 4), whose signalling activities, cellular turnover, trafficking and oncogenic alterations epitomize the complexity of the underlying molecular pathways (Uribe et al. 2021). Among the key events regulating EGFR kinase activity, engagement of intracellular signalling targets and cellular responses is the process of receptor internalization (Tomas et al. 2014). The resulting endosomal containment precedes molecular decisions based on the nature and intensity of the activating signal, or oncogenic alteration of EGFR (Tomas et al. 2014). Consequently, the receptor may be recycled back to the plasma membrane, or redistributed to other subcellular compartments, including microvesicular bodies (MVBs), eventually leading to lysosomal degradation (Tomas et al. 2014). The alternative fate of EGFR involves its extracellular release as cargo of small extracellular vesicles (EVs) originating from intraluminal vesicles formed within the MVB (exosomes) (Sanderson et al. 2008), or in association with larger EVs (Al‐Nedawi et al. 2008) that may be shed directly from the plasma membrane (Di Vizio et al. 2009).

EV‐mediated extracellular emission of the oncogenic EGFR from cancer cells carries unique mechanistic and diagnostic implications (Al‐Nedawi et al. 2008). For example, in glioblastoma (GBM) transfer of the constitutively activated EGFR mutant (EGFRvIII (Furnari et al. 2015)) between cellular populations elicits notable biological responses (Al‐Nedawi et al. 2008), including new forms of vascular growth and accelerated disease progression (Spinelli et al. 2024), among multiple other processes described to date (Song et al. 2016; Frawley and Piskareva 2020; Zanetti‐Domingues et al. 2020).

Mapping EV‐mediated cell‐cell communication pathways involving intercellular transfer of EGFR or other receptors (and macromolecules) in cancer (Al‐Nedawi et al. 2008; Frawley and Piskareva 2020) depends on a better understanding of the nature and diversity among constituent EV subpopulations. This is underscored by the ability of different populations of cancer‐related EVs to interact with different recipient cells (Hoshino et al. 2015) or by observations suggesting that EVs from different cellular sources may ‘compete’ for the same cellular targets (Adnani et al. 2022). Moreover, individual cell types are known to produce varying repertoires of EVs under changing microenvironmental, or stress conditions, with important implications for EV cargo loading and the related biological activity (Montermini et al. 2015; Keklikoglou et al. 2019).

In this regard, relatively little is known about EV landscapes of cancer cells harbouring oncogenic EGFR. EGFR pathway activation may contribute to cancer cell vesiculation, including EGFR export itself, amidst global increase in the emission of EV‐associated proteins (Al‐Nedawi et al. 2008), reprogramming of the expression of genes involved in EV biogenesis and major changes in the EV proteome (Choi et al. 2018). How do these changes affect EV heterogeneity remains poorly understood and dissecting these processes at the single EV level has thus far been limited (Choi et al. 2018; Choi et al. 2019), largely due to accessibility of suitable technologies (Shao et al. 2018).

Here we analyse EV landscapes of GBM cells driven by oncogenic EGFR/EGFRvIII. We document that cancer cells that uniformly express high levels of this transforming receptor on their plasma membranes, release multiple subsets of EVs, some of which are positive for EGFR/EGFRvIII while others are negative. Using nano‐flow cytometry, nano‐chip analysis and super‐resolution nano‐imaging microscopy we document that EGFR‐positive EVs often constitute a minority population within the particulate secretome of EGFR/EGFRvIII‐driven GBM cells, with quantitative estimates varying as a function of technology employed and donor cell characteristics. We document that EGFR‐negative EVs exhibit features partially overlapping with their EGFR‐positive counterparts. The EV mediated release of EGFR can be inhibited by pharmacological agents targeting neutral sphingomyelinase (SMPD3) responsible for exosome biogenesis. However, EGFR loading into EVs is independent of syntenin 1 (SDCBP), which instead regulates the quantitative representations (but not the presence or absence) of pre‐existing EV subpopulations identifiable within the cancer cells secretome by patterns of CD63, CD81 and CD9 co‐expression. Cancer EV subpopulations are readily taken up by microglial cells differentially impacting their protein expression. Overall, our study documents the capacity of different orthogonal nanotechnologies to interrogate the complexity and dynamic heterogeneity within the particulate secretome of EGFR‐driven glioma cells.

## 2 Materials and Methods

### 2.1 Cell Culture

Glioma stem cells (GSC) with mesenchymal characteristics were cultured as described previously (Spinelli et al. 2024). Both wild type (GSC83) cells and their syntenin 1‐edited counterparts (GSC83‐SYN‐KO cells) as well as GSC1123 and GSC1005 cells were grown as spheres in a media containing DMEM‐F12 (GIBCO, 11320033) supplemented with 100 µg/ml EGF (GIBCO, PHG0311L), 100 µg/ml FGF (GIBCO, PHG0261), 0.2% Heparin (STEMCELL, 07980), 1X B27 serum‐free supplement (GIBCO, 17504044), 1% GlutaMAX (GIBCO, 35050061), and 1% penicillin‐streptomycin (GIBCO, 15070063). Established glioma cell lines U373 and U373vIII were cultured as previously described (Magnus et al. 2014) in Dulbecco modified Eagle medium (Wisent, D6429), 10%FBS and 1% Penicillin/Streptomycin. Human embryonic microglial cells (HMC3) were purchased from ATCC (CRL‐3304) and cultured under recommended conditions including EMEM (Wisent, 320‐005) supplemented with 10% fetal bovine serum (FBS; Wisent, 290045) and 1% Penicillin/Streptomycin at 37°C.

### 2.2 Standard Flow Cytometry and Cell Immunostaining

Indicated cells were seeded in 12‐well plates for 24 h, then trypsinized and stained with anti‐EGFR‐Alexa Fluor647‐conjugated antibodies (Biolegend, 352918) and analysed using a BD FACS Canto with FlowJo software version 10.7.1. For confocal microscopy, the adherent cells were fixed with 4% paraformaldehyde in phosphate buffered saline (PBS) for 10 min and, when indicated, permeabilized with 0.1% Triton X‐100 in PBS for 5 min. Both permeabilized and non‐permeabilized cells were then incubated with primary antibodies: anti‐EGFR‐Alexa Fluor647 (Biolegend 352918) and anti‐CD9‐FITC (Biolegend 312104). The cells were subsequently counterstained with DAPI.

### 2.3 Isolation of EVs

GSC cells were cultured in their standard serum‐free growth media, while U373VIII and U373 cells were maintained in medium supplemented with EV‐depleted FBS. After 72 h of culture the conditioned media were collected and centrifuged for 10 min at 300 × *g*, then 20 min at 2000 × *g*, after which the supernatant was filtered through a 0.8‐mm filter (Millipore). The supernatant was then concentrated (centrifuged at 3500 × *g* for 20 min) using Amicon Ultra‐15 Centrifugal Filter Units with 100 KDa molecular cut‐off (Millipore, # UFC905008) to a final volume of 0.5 mL. From this material EVs were pelleted by centrifugation at 100,000 × *g* for 70 min (Beckman TLA100.2 rotor). The resulting EV pellet was resuspended in filtered 1×PBS or RIPA buffer and stored at −80°C (preparation designated for simplicity as 100K).

### 2.4 Nanoparticle Tracking Analysis (NTA) and Transmission Electron Microscopy (TEM)

The size and concentration of EVs were assessed using NTA technology (NanoSight NS500; Malvern Panalytical, Malvern, UK). For this purpose, the samples were diluted with D‐PBS to achieve an optimal concentration of 10^8^–10^9^ particles per mL. The analysis was conducted following the previously described protocol (Spinelli et al. 2024). For TEM analysis, EVs were fixed in a solution containing 2.5% glutaraldehyde in 0.1 M sodium cacodylate (pH 7.4), as described earlier (Tawil et al. 2021). Ten microliters of fixed EV suspension were placed onto an ultrathin carbon film‐coated copper grid. The grid was then rinsed with droplets of deionized water for 15 s, repeated four times, and subsequently stained with 1% uranyl acetate for 45 s. After drying, the morphology of EVs was examined using a transmission electron microscope (Tecnai 12 BioTwin, Philips, Netherlands) operating at 120 kV.

### 2.5 Protein Quantification and Western Blotting (WB)

The total proteins were extracted from cells or EVs using RIPA lysis buffer, supplemented with protease inhibitors (Sigma,11836170001). The extracted proteins were quantified using the Pierce Micro BCA Protein Assay (Thermo Scientific, Rockford, IL, USA). Protein separation was performed using 10% sodium dodecyl sulphate‐polyacrylamide gel electrophoresis (SDS‐PAGE) (BioRad) and subsequently transferred to polyvinylidene difluoride (PVDF) membranes. The membranes were probed with indicated primary antibodies, followed by horseradish peroxidase‐conjugated secondary antibodies (anti‐mouse, Bio‐Rad 1706516; anti‐rabbit, Cell Signalling 7074S). Chemiluminescence was detected using the Amersham ECL WB Detection Kit (RPN2108, GE Healthcare) and visualized using the ChemiDoc MP system (Bio‐Rad). Primary antibodies included rabbit anti‐CD81 (Cell signalling, 56039), rabbit anti‐CD9 (Abcam, ab223052), rabbit anti‐CD63 (Abcam, ab134045), Alix (Abcam ab186429), anti‐CD44 (Abcam, ab157107), rabbit anti‐syntenin (Abcam, ab133267), mouse anti‐actin (Sigma A1978).

### 2.6 Nano‐Flow Cytometry

Nano‐flow cytometry was carried out using the CytoFLEX system (Beckman Coulter, Pasadena, CA). GSC83‐EVs were incubated with specified fluorophore‐labelled antibodies for 2 h at room temperature in the dark. Isotype controls corresponding to the respective antibodies were included in all experiments. All fluorophore‐labelled antibodies, including anti‐CD9 (APC) and EGFR (Alexa488) were obtained from Biolegend (cat#352907). The instrument calibration, gating samples and signal acquisition were performed as per recommended guidelines and earlier publications (Choi et al. 2019).

### 2.7 ExoView Analysis

ExoView chips pre‐coated with anti‐CD63 (clone H5C6), anti‐CD81 (clone JS81), or anti‐CD9 antibodies (clone H19a) were used to capture corresponding EV subpopulations, as per manufacturer protocols (Nanoview Biosciences). Mouse IgG1κ matching isotype antibody was used as a negative control for non‐specific EV binding to chips that were pre‐scanned following the manufacturer's instructions to establish the baseline measurements before sample incubation. A total of 40 µL of pre‐diluted EV preparation was carefully applied to the pre‐scanned chip and incubated overnight at room temperature in a sealed 24‐well plate to prevent evaporation. The next day, the captured GSC‐EVs were stained with anti‐CD63 (CF‐647), anti‐CD81 (CF‐555), and anti‐EGFR (Alexa488) antibodies (Biolegend, 352907) according to the manufacturer's protocol. The anti‐tetraspanin antibodies on the chips and those used for fluorescence detection were identical clones. All reagents were stored at 4°C and brought to room temperature before use, and all incubation steps were carried out with gentle shaking (350 rpm on an orbital shaker). First, the chips were immersed in 1000 µL of Solution A (NanoView Biosciences), after which 750 µL were aspirated and replaced with fresh solution A. This wash cycle was repeated three times, with a 5‐min incubation between each. Simultaneously, an antibody detection mixture was prepared by diluting anti‐EGFR (ALX488), anti‐CD63 (CF‐647), and anti‐CD81 (CF‐555) antibodies 1:500 in blocking buffer (both from NanoView Biosciences). After the third solution A removal, 250 µL of the antibody mixture was added and incubated for 1 h at room temperature. To minimize fluorophore bleaching, all subsequent steps were performed under aluminium foil cover. Unbound antibodies were washed out by adding 500 µL of solution A, bringing the total volume back to 1000 µL, followed by three more wash cycles with solution A as previously described. Excess detergent was removed by washing three times with Solution B (NanoView Biosciences). Finally, 750 µL of solution B were aspirated and replaced with 750 µL of Milli‐Q water. The chips were carefully removed from the well with tweezers and submerged in Petri dishes filled with Milli‐Q water, ensuring the antibody spots remained wet. The chip was gently swirled in the water and then slowly lifted from the Milli‐Q bath at a 45° angle, allowing surface tension to dry it. The chips were placed on lint‐free Kimtech wipes (Kimberly‐Clark) to absorb the remaining water and then moved to the sample stage for fluorescent and interferometric imaging. Images acquired were analysed using ExoViewer 2.5.0 software, with EV sizing thresholds set to 50–200 nm diameter. The percentage of EGFR, CD63 and CD81 expressing EVs in the anti‐CD9 spot was calculated and graphed.

### 2.8 Super‐Resolution Microscopy

EVs were immunolabeled and imaged using the EV Profiler Kit (#EV Profiler 1 application kit, ONI) by employing direct stochastic optical reconstruction microscopy (dSTORM) technique. Briefly, approximately 1.0 × 10^8^ EVs were immobilized in a 100 µL volume chamber, which was 3D‐printed using a digital light processing (DLP) printer and adhered to coverslips treated with poly‐L‐lysine. After fixation via ONI EV Profiler 1 fixing buffer, excess EVs were washed, and the chambers were treated with blocking agents (ONI EV Profiler 1 application kit). The samples were incubated for 1 h at 37°C with 30 µL of anti‐EGFR‐AF488 antibody (Alexa488; Biolegend, 352907) diluted 1:100 in sterilized water. Samples were further incubated with 30 µL of anti‐CD81‐ONI647 diluted 1:10 and 30 µL of anti‐CD63‐ONI568 diluted 1:10, both in sterilized water. These antibodies were provided by ONI as part of the EV Profiler 1 application kit, along with the blocking agent B1. Finally, samples were again fixed with F1 for 10 min, and freshly prepared dSTORM‐imaging buffer was added prior to image acquisition. Using the Nanoimager microscope (ONI; Oxford Nanoimaging, UK), equipped with a 100×, 1.4 NA oil immersion objective lens, with the angle of illumination set to 52° for total internal reflection fluorescence (TIRF) imaging. Before image acquisition, 100 nm Tetraspec beads (Thermo Fisher Scientific) were used in two fluorescent channels for colocalization calibration, following MIFlowCyt‐EV guidelines (Welsh et al. 2020; Lee et al. 2008). AF488‐conjugated anti‐human CD9 and AF647‐conjugated anti‐human CD81 were illuminated using 488 and 647 nm lasers, respectively. For each channel, 2000 frames were recorded, resulting in a total of 6000 frames across the three channels. The exposure time was set to 30 ms per frame. Co‐localization analysis of two or three different molecules on individual EVs was conducted using the CODI online analysis platform. Drift correction was applied across all 6000 frames to ensure accurate localization. Temporal grouping for quantification was performed with a frame gap of 2 and a maximum inter‐molecular distance of 30 nm. This maximum distance parameter, which was varied between 20 and 60 nm during optimization, was finalized at 30 nm to achieve the best compromise between spatial resolution and accurate identification of EV particles, accounting for EV size, fluorophore properties, and molecular density. Localization clusters containing more than 15 individual molecules were classified as EV clusters. Clusters were further refined based on shape, with skewness values between 1 and 2.9 and circularity (Circ) between 0.7 and 1 used as selection criteria. An object was identified as a single EV if more than 5 individual molecules were detected in the same channel within a radius of 100 nm from the centre of the cluster. Five fields of view were acquired from each sample for statistical analysis. To control for false positives, images were acquired from bare coverslips modified with poly‐L‐lysine and incubated with negative control antibodies (AB 647 and AB 488). False positive detections in these control images were fewer than 240 and 30 particles per image for the respective channels, and these were excluded from subsequent analyses. The EV populations were computed from images using the CODI software.

### 2.9 Immuno‐Separation and Proteomic Profiling of EGFR‐Positive and EGFR‐Negative EVs

Dynabeads Protein G magnetic beads (ThermoFicher, Catalog No. 10003D) were conjugated with EGFRvIII antibody (Kerafast, Anti‐EGFRvIII [L8A4], EDK002). For this purpose, 1.5 mg (equivalent to 50 ul) of beads was incubated with 3 ug of EGFRvIII antibody (equivalent of 3 ul) in PBS overnight at 4°C, with rotation. Next day, the beads were washed 3 times with PBS using the magnetic rack, and resuspended in fresh PBS. To capture EGFRvIII expressing EVs, 10 ug of freshly isolated total GSC83‐EV preparation was incubated with anti‐EGFRvIII‐conjugated beads in a maximum volume of 200 ul in PBS and incubated overnight at 4°C, with rotation. The following day, beads coated with EVs, enriched for EGFR protein (EGFR‐positive EVs) were isolated from the remaining EV flow‐through fraction (EGFR‐negative EVs) using magnet. The isolates of EGFR‐positive (beads) and EGFR‐negative EVs (pellet) were then lysed in RIPA buffer for protein extraction and analysis including mass spectrometry and immunoblotting. WB validation of effective EV enrichment/depletion for EGFR was conducted as described earlier. To extend this analysis to the whole proteome four biological replicates were prepared for each sample type. Equal amounts of protein (1 µg per sample) were subjected to common sample processing and analysed using a high resolution Orbitrap Astral Mass Spectrometer, according to the protocol detailed below. Data analysis was performed using the DIA‐Analyst online tool. Filtering criteria included VSN normalization, Perseus‐type imputation, and Benjamini–Hochberg false discovery rate (FDR) correction. Data were appended as Table S2 (significantly changed protein expression) and Table S3 (all proteins). A log_2_ fold change cutoff of 1 and an adjusted *p*‐value cutoff of 0.05 were applied. The results were visualized using volcano plot, box plots, and heat map. Gene Ontology (GO) enrichment analysis of differentially expressed proteins was conducted using ShinyGO (v0.85.1) with an FDR cutoff of 0.05. Kyoto Encyclopedia of Genes and Genomes (KEGG) pathway enrichment analysis was performed using DAVID bioinformatics tool. Graphs of enriched biological pathways for EGFR‐positive and ‐negative EVs were generated using MATLAB software. The selected protein hits were further validated by Western blot, as depicted in corresponding figures.

### 2.10 Generation of Syntenin 1‐Deficient Glioma Stem Cells

Syntenin 1 (SDCBP) deficient glioma stem cells (GSC83) were generated using CRISPR/Cas9 gene editing approach using lentiviral vectors. For lentivirus production the day before transfection, 4.5 × 10^6^ 293T packaging cells were seeded onto 10 cm dish (9 mL). On the day of transfection, the plasmid mixture: VSV‐G (8454 Addgene), pRRE (12251 Addgene), REV (12253 Addgene) and transfer plasmids were added. As transfer plasmids we used sgRNAs in pCLIP‐Dual‐SFFV‐ZsGreen for SDCBP CRISPR guides (TEDH‐1070533, TEDH‐1070531, TEDH‐1070532 Transomic) and pCLIP‐Cas9‐Nuclease‐hCMV‐tRFP (SHB_2264 Transomic) for the CAS9 activity. The guide RNA plasmid and CAS9 plasmid were obtained from Dr. Sidong Huang, McGill University. For transfection 2 x HBS buffer was mixed with the solution containing 2 M Calcium and the plasmid mixture as mentioned above. Cells were then incubated overnight (16–18 h) at 37°C, 5% CO2. On the next day the media was collected, and cell debris was removed by centrifugation at 1500 × *g* for 10 min (4°C). The remaining supernatant was used to recover the pellets of lentiviral particles by centrifugation at 22,000 rpm for 2 h, after which the pellets were re‐suspended in 50 uL of PBS. CRISPR‐CAS 9 gene editing to produce SDCBP gene knock‐out in GSC83 cells was performed by lentiviral transduction, with the vector described above. Cells were first transduced with CAS9‐RFP containing virus for 16 h. Positive cells were then sorted by flow cytometry, after which RFP positive cells were transduced with three different viruses (each of them carrying two guide RNAs and GFP cassette). After 4 washes with PBS the cells were cloned using FACS, expanded, permeabilized and stained with APC‐conjugated anti‐human SDCBP antibody (Abcam ab133267). Single clones that were positive for both RFP and GFP, but negative for APC were then validated by WB, which revealed the absence of SDCBP protein expression. The reagents used were as follows: Plasmid 1 TEDH‐1070533: Guide 1: ATGGTGAAAACTGTGCAGGA; Guide 2: CATAACATCTGTGAAATCAA; Barcode: TAATCTTGGAAGCCCTATCTATCG; Plasmid 2 TEDH‐1070531: Guide 1: ATAAACCTACTTCCATCGTG; Guide 2: CGTAGAGCAGAAATTAAGCA; Barcode: GCTTTTGAGGCTAGGCAGGGGGGG; Plasmid 3 TEDH‐1070532: Guide 1: TCTGCTCCTATCCCTCACGA; Guide 2: TATATTTGTTCAGCTAGTCC; Barcode: TAGATTGCAGAAAGGTCCATCAGG.

### 2.11 Treatment With Neutral Sphingomyelinase Inhibitor

To block the activity of neutral sphingomyelinase‐dependent EV biogenesis pathway the cells were treated with GW4869 inhibitor. The viability of GSC83 cells was evaluated in the presence or absence of GW4869 or DMSO and found unaffected across a wide spectrum of concentrations. GSC83 cells were seeded in 96‐well plates in complete growth media and incubated for 24 h. The next day, cells were washed and treated with varying concentrations of GW4869. They were then maintained in complete growth media for the entire duration of the assay. Absorbance at 490 nm was measured at specified time intervals, with the signal indicating viable cell numbers assessed for up to 3 days post treatment. Subsequently, GSC83 cells were treated with 50 µM GW4869, and after 72 h, the culture supernatant was collected, and EVs were isolated and analysed as described.

### 2.12 Analysis of Cellular EV Uptake Using Confocal Microscopy

Human microglial cells (HMC3) were plated on μ‐Slide 8‐Well ibiTreat chambered coverslips (ibidi, Germany) at a density of 5 × 10^3^ cells per well and incubated overnight. HMC3 cells were then incubated with GSC83‐EVs (35 µg) pre‐labelled with fluorescent dye 124‐carboxyfluorescein succinimidyl ester Far Red (CFSE) (Thermo Fisher, C34572) for 24 h. The cell nuclei were counter‐stained with DAPI (4′,6‐diamidino‐2‐phenylindole) for fluorescence visualization. Images were captured using an LSM780 confocal microscope (Carl Zeiss, Thornwood, NY, USA) equipped with a 63× objective.

### 2.13 Mass Spectrometry of EV Recipient Cells

The molecular responses of microglial HMC3 cells following uptake of EVs from cancer cells were assessed by analysis of protein profiles using mass spectrometry, as described earlier (Choi et al. 2018) in three independent biological repeats. HMC3 cells were seeded at 3 × 10^5^ cells/well in six well cluster plates, to each of which EVs from indicated GSC83 cultures were added at 100 µg of EV protein equivalents. EVs were isolated from GSC83 cell culture media of cells treated with GW4869 at 50 µM for three days or corresponding amount of diluted vehicle (DMSO) in PBS. The untreated control wells received PBS alone. Upon treatment cells were lysed, protein was extracted and 10 µg aliquots were desalted using SDS‐PAGE, loaded onto the stacking gel, and stained. Destaining was done for each of the three biological replicates prior to analysis by LC‐MS/MS. Dithiothreitol (DTT) was used to achieve reducing conditions for the in‐gel trypsin digestion, and iodoacetic acid was used to accomplish alkylation as previously described (Choi et al. 2018). Following this step, 0.1% aqueous formic acid 2% acetonitrile was used to re‐solubilize the lyophilized peptides, the peptides were then placed onto a 75 µM Thermo Acclaim Pepmap ID 2 cm C18 3 µM beads before applying to Acclaim Pepmap Easyspray (Thermo, 75 µM Å∼ 15 cm) column. Analytical column separation was carried out with two µMC18 beads using a Dionex Ultimate 3000 uHPLC at 220 nL/min over 2 h with a gradient of 2%–35% of organic solvents (0.1% formic acid in acetonitrile). Peptides were analysed using a Orbitrap Fusion Tribrid mass spectrometer (Thermo Fisher Scientific) operating at 120,000 resolutions (FWHM in MS1, 15,000 for MS/MS) with higher‐energy collisional dissociation sequencing of all peptides with a charge of 2+ or greater. The raw data were converted into *.mgf format (Mascot generic format) and searched using Mascot 2.3 against human sequences (SwissProt). The database search results were loaded onto Scaffold Q+ Scaffold 5 (Proteome Sciences) for spectral counting, statistical treatment and data visualization. Protein threshold was ≥ 0.99% and peptide threshold ≥ 0.95% with 2 minimal peptides. These selected proteins were quantified by label‐free protein quantitation integrated into Scaffold 5, employing the following options: use normalization, minimum value (0.0), and Quantitative Method (Weighted Spectra). Proteins with less than 0.05 of *p*‐value were considered as significantly changed. The results were analysed using One‐way analysis of variance (ANOVA) using scaffold version 5, and out of 100 top proteins with the highest level of significance a subset of 46 top proteins with the highest fold difference between DMSO and GW4869 groups were selected ranked and visualized as heatmap using clustvis software. The colours in the heatmap depict the quantitative expression of indicated proteins as measured by normalized weighted spectra. A complete list of detected proteins is provided in Table S3. Lists of identified proteins were imported into the DAVID Bioinformatics database (davidbioinformatics.nih.gov) and assigned to their GO and KEGG pathway annotations. Validation was performed by immunoblotting for EGFR with indicated conditions (Figure S6) to control for potential residue of GW4869 in EV preparations.

### 2.14 Data Analysis and Statistics

All experiments were independently reproduced at least three times unless otherwise indicated, and quantitative data were plotted as mean ± standard deviation (SD). Data were analysed for statistical significance using an unbiased Student's t‐test for comparisons between two groups, and one‐way ANOVA was employed for comparing multiple groups. All analyses were performed using GraphPad Prism v9 or Excel packages. Proteomic datasets are appended for EVs separated using magnetic beads (Table S1 – significant changes; Table S2 – all proteins) and for EV‐treated HMC3 cells (Table S3). Statistical significance was determined with *p* ≥ 0.05 indicating no significant difference (NS), while asterisks denoted significance: *—at *p* < 0.05, **—at <0.01, ***—at <0.001 and ****—at <0.0001.

## 3 Results

### 3.1 Pronounced and Uniform Surface Expression of Oncogenic EGFR Among Subsets of Glioma Cells

**FIGURE 1:**
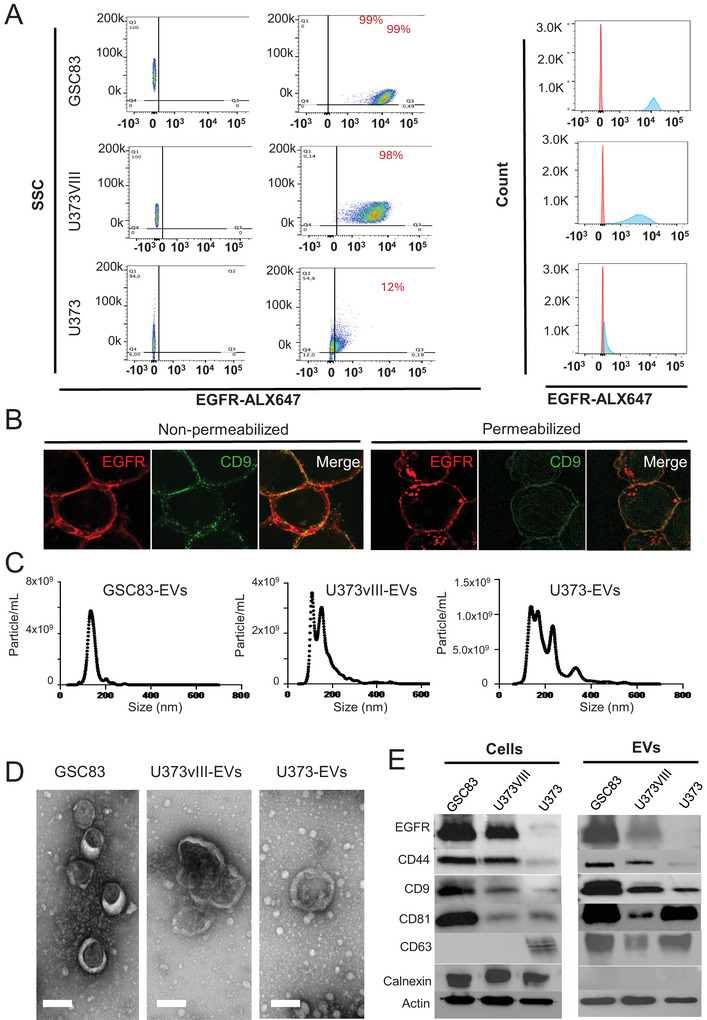
Human glioma cells exhibit variable but uniform cellular levels of EGFR, which is released as cargo of extracellular vesicles. (A) Flow cytometry analysis illustrating the uniform presence of EGFR antigen on the surface of glioma cells with high (GSC83 and U373VIII) and low (U373) levels of EGFR/EGFRvIII expression (right panel—dot plot; left panel—histogram); (B) Confocal imaging of immunofluorescent staining for EGFR antigen (red) and CD9 (green) in adherent GSC83 cells either intact or permeabilized before antibody exposure. EGFR and CD9 co‐localize mainly around plasma membrane (size bar—20 µm). (C) Nanoparticle tracking analysis (NTA) reveals comparable extracellular vesicles (EV) profiles of conditioned media between indicated glioma cell lines. (D) Transmission electron microscopy (TEM) depicting comparable EV morphology between secretomes of GSC83, U373VIII and U373 cells (scale bar—100 nm). (E) Immunoblotting for indicated protein markers reveals the presence of EGFR in EVs of EGFR‐expressing cells and some variability in protein composition among glioma cell derived EVs (see text for details).

Within GBM diagnosis different disease subgroups vary with respect to molecular make up and EGFR status (Wen et al. 2020; Neftel et al. 2019). This complexity is further compounded by cellular heterogeneity within individual tumours, including variable levels of EGFR/EGFRvIII (Furnari et al. 2015) a landscape conducive of emerging molecular gradients and intercellular exchange of these RTKs as cargo of EVs (Al‐Nedawi et al. 2008). While GBM cells and tumours are known to shed and uptake EGFR/EGFRvIII‐positive EVs (Al‐Nedawi et al. 2008; Graner et al. 2009) it is unclear whether packaging of this receptor into EVs is obligatory for EGFR/EGFRvIII positive cancer cells. In order to glean some related insights, we used conventional flow cytometry to test EGFR antigen expression on the surfaces of individual glioma cells that were either weakly positive for the wild type EGFR (U373), or were engineered to overexpress oncogenic EGFRvIII (U373vIII) (Micallef et al. 2009). We also compared them to mesenchymal glioma stem cells (GSCs) endogenously expressing high levels of EGFR and EGFRvIII (GSC83) (Spinelli et al. 2024; Mao et al. 2013). The representative dot plots and histograms depicted in Figure 1A illustrate the expected (Spinelli et al. 2024; Magnus et al. 2014; Al‐Nedawi et al. 2010) levels of EGFR/EGFRvIII expression by these respective cell lines and suggest that while few (12%) U373 cells harbour low levels of this receptor on their surfaces, both U373vIII and GSC83 cells are highly and uniformly positive for EGFR/EGFRvIII (98% and 99%, respectively). The expression of EGFR/EGFRvIII antigen was not only ubiquitous among GSC83 cancer cells, but also evenly distributed around the plasma membranes with considerable overlap with CD9 tetraspanin which is typically enriched in EVs. This proximity is revealed by confocal images of these cells co‐stained for EGFR and CD9 either with or without membrane permeabilization (Figure 1B). Thus, plasma membranes of EGFR‐positive glioma cells, especially their mesenchymal subtype (GSC83), appear to be strongly and uniformly decorated with this receptor, with a pattern comparable to that of CD9 tetraspanin. This spatial distribution raises the question as to whether the abundance of EGFR/EGFRvIII in plasma membranes of cancer cells would result in their ability to produce predominantly EGFR/EGFRvIII‐positive EVs (Al‐Nedawi et al. 2008; Spinelli et al. 2024).

### 3.2 EV Profiles of Glioma Cells Expressing Different Levels of EGFR

Irrespectively of their EGFR status glioma cells release ample amounts of EVs with predominant sizes of 100–150 nm as determined by NTA (Figure 1C) and TEM (Figure 1D). The bulk analysis of EV‐associated proteins revealed the expected abundance of EGFR in EVs (and in cell lysates) of GSC83 and U373vIII cells along with the corresponding enrichment for CD44, a marker of aggressive, mesenchymal and glioma stem cell‐related phenotype (Mao et al. 2013). Small EVs from all cell lines also expressed canonical tetraspanins (CD63, CD81 and CD9) with some variability in levels, but they were all negative for calnexin (CANX) suggesting their acceptable purity (Welsh et al. 2024) (Figure 1E). These results are in agreement with previously reported data suggesting a robust vesiculation associated with EGFR/EGFRvIII driven glioma cells (Spinelli et al. 2024; Choi et al. 2018; Choi et al. 2019).

### 3.3 Heterogeneous EGFR Expression Among Glioma EV Subpopulations

**FIGURE 2:**
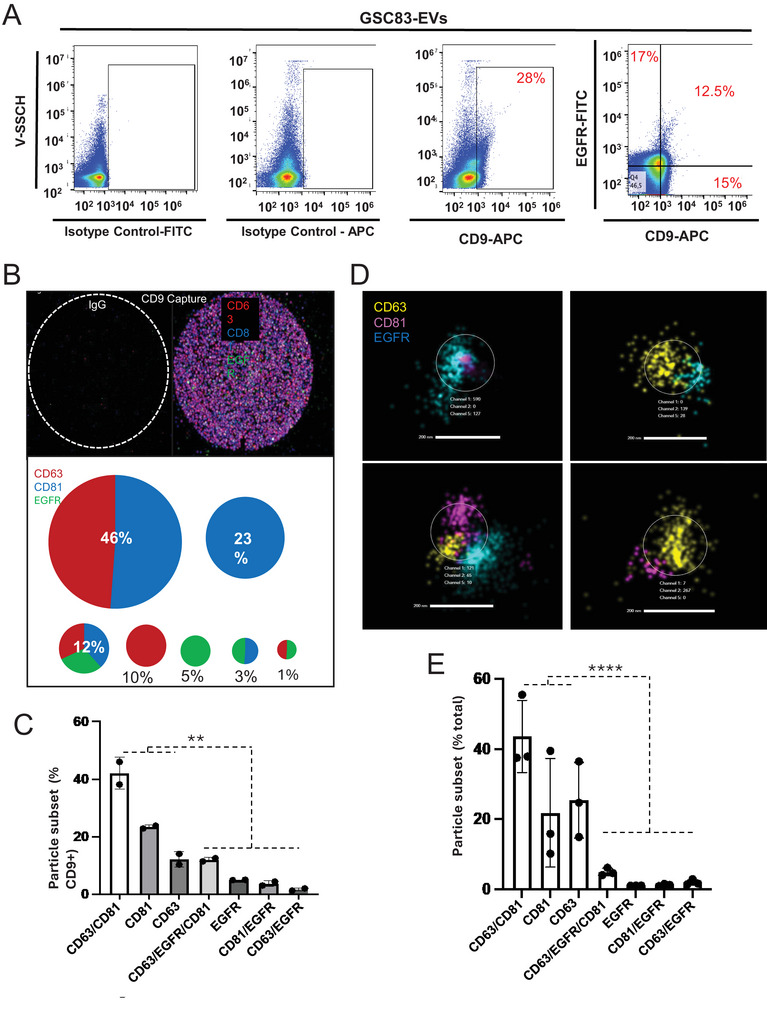
Molecular heterogeneity of EV subpopulations released by EGFR‐expressing glioma cells. (A) Nano‐flow cytometry mapping of CD9 and EGFR expressing subpopulations of GSC83‐derived EVs (positive regions were gated against PBS background); (B) ExoView R100 chip analysis of EV subpopulations with different expression profiles of EGFR, CD63 and CD81 following capture on immobilized anti‐CD9 antibody (percentages reflect the fraction of the total particle count assigned to each EV phenotype); (C) Quantification of ExoView analysis including two independent repeats reveals only a small fraction of GSC83‐derived EVs that contain EGFR amidst seven different EV populations defined by the configuration of three markers used (EGFR, CD63, and CD81); (D) Super‐resolution dSTORM analysis (ONI) of GSC83‐derived EVs as defined by patterns of EGFR, CD63 and CD81 colocalization (images of individual EVs with different phenotypes); (E) Quantification of ONI analysis including three independent repeats reveals that a minority of GSC83‐derived EVs contain EGFR amidst seven EV phenotypes distinguished by three markers used (EGFR, CD63, CD81) *p* values: NS—> 0.05; *—< 0.05; **—< 0.01; ***—< 0.001; ****—< 0.0001 (see text for further details).

Because patient‐derived GSC83 cells endogenously express high and uniform levels of EGFR/EGFRvIII and release EGFR‐enriched EVs they could be especially informative as to processes that control EV biogenesis and EGFR release in glioma. For these reasons we further dissected the EV repertoire of these cells by nano‐flow cytometry with single vesicle resolution (Figure 2A). Surprisingly, while the aforementioned bulk immunoblot analysis revealed a robust EGFR protein content associated with small EVs (Figure 1E) only a fraction (∼30%) of GSC83‐EVs stained positive for this antigen in CD9/EGFR co‐staining experiments, with 12.5% of them expressing CD9/EGFR double positivity. While nano‐flow cytometry exhibits known challenges (Choi et al. 2019) and additional quantitative improvements could render our estimates more precise (Welsh et al. 2020), our main observation suggests that a large percentage of GSC83‐EVs may not express EGFR (or express it at very low levels) and in some cases EV‐associated EGFR may be decoupled from specific tetraspanins (e.g., CD9 in this case) often present in membranes of canonical small EVs (Kowal et al. 2016).

To further extend and validate these results we employed an orthogonal, high through‐put chip‐based single EV technology (ExoView) where thousands of EVs can be immobilized on functionalized surfaces, labelled with three different fluorescent probes and quantified. Indeed, when GSC83‐EVs were captured using anti‐CD9 antibody and stained for EGFR, CD63 and CD81 the pattern of co‐expression of these markers revealed at least seven different EV subpopulations (Figure 2B,C). Among them four different CD9+ EV subsets contained detectable EGFR, which together accounted for approximately 21% of the total EV output. Notably, while the largest EV subpopulation (CD81+/CD63+) comprising 46% of all CD9+ EVs did not contain EGFR, approximately 5% of EVs negative for both CD63 and CD81 contained EGFR (Figure 2C). This heterogeneity may suggest the existence of diverse molecular processes leading to EV cargo assembly, including pathways that control vesicular emission of EGFR either in the presence or in the absence of common CD63/CD81 tetraspanins (Varn et al. 2022).

As an alternative to the aforementioned, CD9‐mediated EV capture‐based technology we also used an unbiased approach relying on immobilization of all EVs on activated surfaces followed by staining for EGFR, CD81 and CD63 with subsequent nano‐imaging (ONI) in dSTORM mode (Figure 2D). This approach, which also enables quantification of EV subpopulations (Figure 2E) revealed that only approximately 9% of all EVs contained EGFR, and the largest contribution to the vesicular secretome of GSC83 cells was, again, from the CD63/CD81 double positive and EGFR‐negative EV population. While different single EV technologies used in these experiments exhibit considerable numerical differences (2‐3 fold discrepancy) in detecting EGFR‐carrying EVs they all capture the fact that this oncoprotein, which is highly abundant on cancer cell surfaces and known to be processed through endosomal pathway (Tomas et al. 2014) contributes to only a small fraction of cancer‐derived EVs. Moreover, these EVs exhibit considerable molecular heterogeneity even with limited numbers of markers used in our experiments. Naturally, these findings also raise the question as to what are the biological roles, biogenetic origins, regulation and functional properties of glioma (cancer) EVs that are either enriched (Al‐Nedawi et al. 2008; Spinelli et al. 2024), or depleted for the EGFR protein cargo?

It should also be noted that the absence of EGFR in a large proportion of GSC‐derived EVs applies to several different EGFR‐expressing mesenchymal glioma stem cell isolates including GSC83, GSC1123 and GSC1005 used in our studies ((Spinelli et al. 2024) Figure S1). However, the degree to which EGFR is packaged into GSC‐EVs may vary between donor cells from as low as approximately 20% to nearly 49%, as revealed by our comparative ExoView analysis (Figures 2 and S1). This diversity may reflect the levels of available EGFR in donor cells (Garnier et al. 2018), as well as differences in regulatory mechanisms modulating the process of EGFR loading among specific cellular subsets.

### 3.4 Distinct Characteristics of EGFR‐Positive and ‐Negative Glioma EVs

**FIGURE 3:**
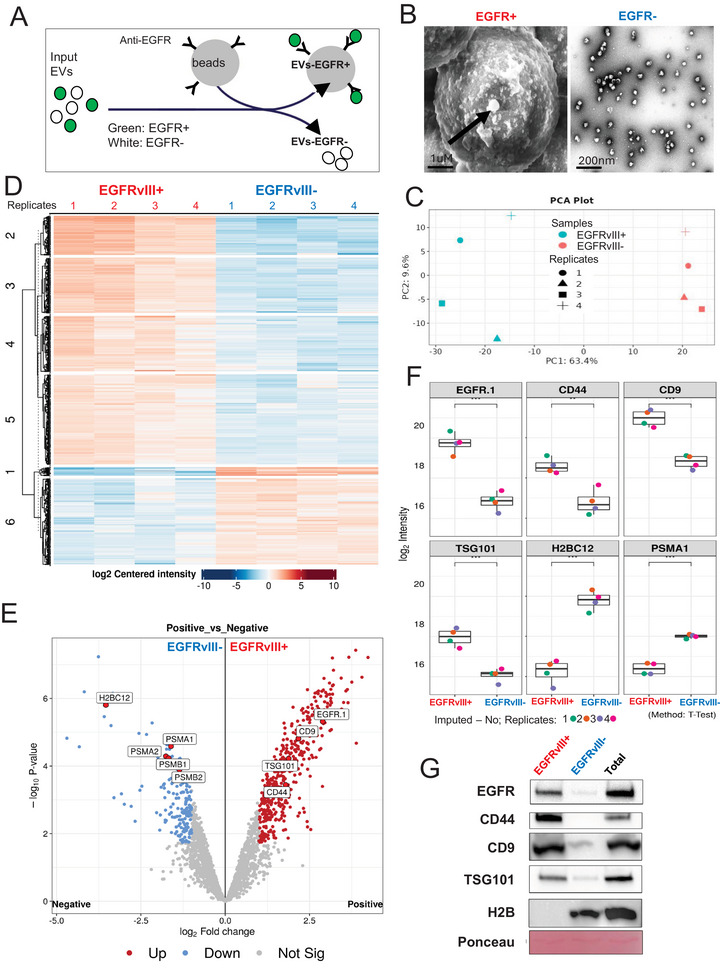
Isolation and proteomic profiling of EGFRvIIII‐positive and EGFRvIII‐negative GSC83‐derived EV subpopulations. (A) Schematic of EV separation using magnetic beads conjugated with anti‐EGFR antibodies. (B) Scanning electron micrograph (SEM) or GSC83‐derived EVs captured on anti‐EGFR‐coated magnetic beads (left panel) and TEM (right panel) of their EGFR‐negative counterparts present in the flow‐through fraction (size bars: 1 µm and 200 nm, respectively); (C) Principal component analysis (PCA) showing global proteomic segregation between EV samples captured by magnetic beads coated with anti‐EGFRvIII antibody (EGFRvIII‐positive) and their ‘flow through’ (EGFRvIII‐negative) counterparts. Each dot represents an individual biological sample. (D) Heatmap depicting differentially expressed proteins (DEPs) identified by high resolution mass spectrometry. The heatmap demonstrates hierarchical clustering of samples according to EGFR status with respective subsets of proteins being either enriched or depleted. (E) Volcano plot illustrating quantitative enrichment between EGFRvIII‐positive (red) and EGFRvIII‐negative (blue) groups of proteins identified by mass spectrometry. Significantly upregulated and downregulated proteins were identified by coloured dots. Analysis was performed via DIA‐Analyst with Benjamini Hochberg as type of FDR correction and adjusted *p*‐value at 0.05. (F) Box plots showing relative abundance levels of selected significantly altered proteins in EGFRvIII‐positive versus EGFRvIII‐negative EV samples. The expected enrichment for EGFR peptides in EVs captured on anti‐EGFRvIII coated beads corresponds to similar enrichment of mesenchymal (CD44) and canonical EV markers (CD9, TSG101), while EGFRvIII‐negative EVs exhibited the increased presence of chromatin (H2BC12) and proteasome (PSMA1) proteins; (G) Western blot validation of representative differentially expressed proteins identified by mass spectrometry analysis.

In order to further ascertain whether EGFR is associated with some specific subsets of EVs, or randomly (if incompletely) assigned to different EV subpopulations, we physically separated EGFR‐positive and ‐negative GSC83‐EVs using magnetic beads, as described earlier (Spinelli et al. 2024) (Figure 3A,B). We extensively depleted GSC83‐EV preparations using magnetic beads pre‐coated with anti‐EGFRvIII antibody until almost no EGFRvIII protein signal could be detected in the flow‐through fraction upon removal of the beads. While several protocols were tested during these experiments with different anti‐EGFR antibodies, anti‐EGFRvIII‐based separation was chosen over total EGFR due to cancer specificity of the EGFRvIII receptor, its frequent co‐expression with wild type EGFR and its abundance in GSC83 cells as well as in their total EV preparations. Importantly, this choice was also informed by a better performance of the respective anti‐EGFRvIII antibodies. The electron microscopy images of the beads coated with EVs versus unbound EVs remaining in the flow through supernatant document the presence and morphology of the respective particle populations (Figure 3B). Each of the resulting EV fractions was then subjected to high resolution mass spectrometry (Figure 3D–F) followed by immunoblot analysis of selected differentially expressed proteins (Figure 3G). As expected, proteomes of EGFRvIII+ and EGFRvIII‐ EVs clustered separately in principal component analysis (PCA) plots (Figure 3C) and several proteins were selectively enriched or depleted in the respective EV subpopulations as indicated by the heat map (Figure 3D). A deeper quantitative analysis of these bulk EV datasets revealed not only some commonalities, but also significant differences between EGFRvIII+ or EGFRvIII‐ glioma EVs as indicated by volcano plot (Figure 3E). Notably, while EGFR, CD44, along with canonical EV‐associated CD9 and TSG101 peptides were highly represented in EGFRvIII+ preparation, the EGFRvIII‐ EVs were enriched in proteasome components (PSMA1, PSMA2, PSMB1, PSMB2) and histones, suggesting contribution of different subcellular compartments (Figure 3E,F). It is possible that EGFRvIII‐negative vesicular fraction visualised in these experiments may also contain non‐vesicular particles, or unique EVs enriched in proteasome as recently described (Kim et al. 2025), or EVs loaded with chromatin (Wortzel et al. 2024). Some of the differences between EGFRvIII‐positive and ‐negative particulates were also independently visualized using immunoblotting (Figure 3G). A more global analysis of these datasets using GO terms revealed enrichment in junctions, adhesion, endocytosis and signalling pathways in EGFRvIII+ EVs, which were also enriched in corresponding molecular functions such as G protein signalling, cadherin binding or integrin binding, among others (Figure S2A,B). In contrast, proteomes of EGFRvIII‐ EV/particles were associated with GO terms, such as: wound healing, regulation of cell migration, cell motility, while the key molecular functions associated these EVs (particles) included metabolic and chromatin binding categories (Figure S3A,B). Kyoto Encyclopedia of Genes and Genomes (KEGG) pathway analysis suggested enrichment of EGFRvIII+ EVs for adhesion‐related processes (Figure S4), while for EGFRvIII‐ fraction the predominant feature was the proteasome pathway (Figure S5; Tables S1 and S2). Whether these properties of EGFRvIII‐positive or ‐negative EVs/particles are relevant for the biology of EGFR/EGFRvIII‐driven GBM remains to be studied. However, these experiments support the notion that EGFR/EGFRvIII tracks with molecularly different EV subpopulations then those that are EGFR/EGFRvIII‐negative, even if both come from the same EGFR‐positive cellular source. Notably, this quantitative bulk EV analysis reflects a different dimension of the particulate secretome than profiling at single EV level.

### 3.5 Release of EGFR‐Carrying GBM EVs Is Independent of Syntenin1

**FIGURE 4:**
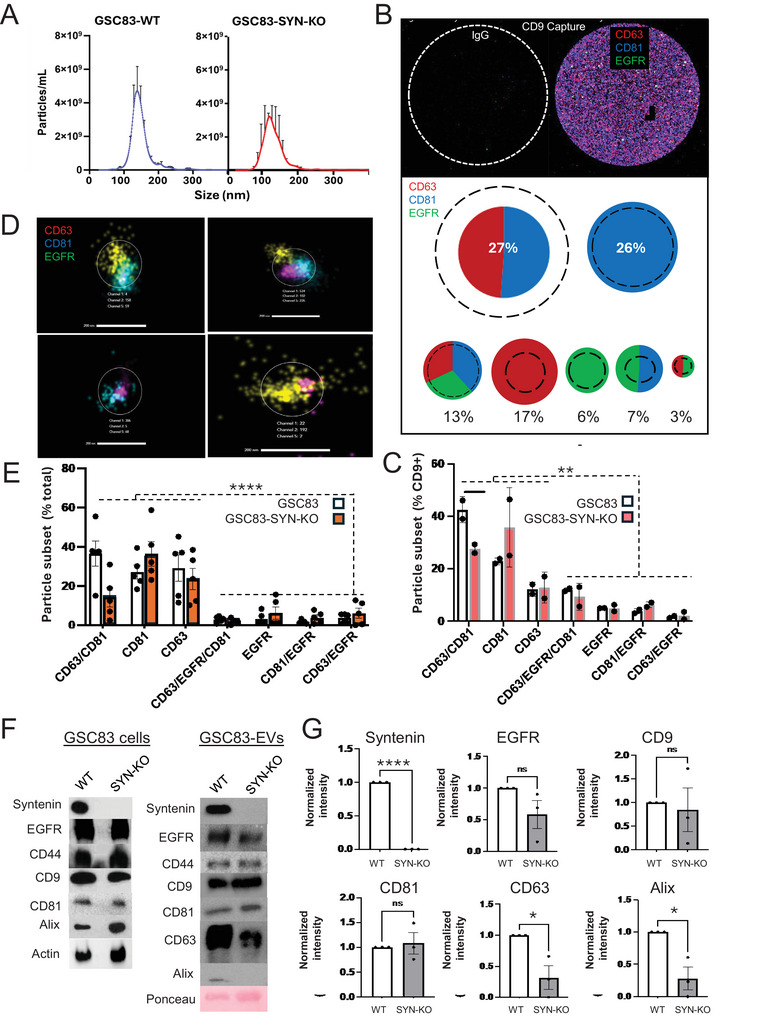
Depletion of syntenin 1 does not abolish EGFR packaging into glioma EVs but modulates their repertoires. (A) NTA profiles reveal a modest impact of syntenin 1 (SDCBP) gene disruption in GSC83 glioma stem cells on characteristics of EVs released into conditioned media after 72 h (*n* = 3); (B) ExoView R100 chip analysis of EV subpopulations released by syntenin 1‐disrupted GSC83 cells (GSC83‐SYN‐KO) in comparison to EVs of wild type GSC83 cells (dashed circles, data from Figure 2B). Individual EVs were captured on anti‐CD9 coated chips and labelled with fluorescent antibodies against EGFR, CD63 and CD81 (percentages reflect the fraction of the total particle count assigned to each EV phenotype); (C) Quantification of ExoView analysis including two independent repeats reveals that a small fraction of GSC83‐derived EVs contains EGFR amidst seven different EV populations defined by three markers used (EGFR, CD63, and CD81). Comparisons between EVs from wild type GSC83 EVs (white bars) and GSC83‐SYN‐KO EVs (red bars) document modest changes in abundance of EV subpopulations; (D) Super‐resolution dSTORM analysis (ONI) of GSC83‐SYN‐KO‐derived EVs as defined by patterns of EGFR, CD63 and CD81 colocalization (images of individual EVs with different phenotypes); (E) Quantification of ONI analysis including five independent repeats reveals that a minority of EVs derived from GSC83 cells contains EGFR, regardless of the syntenin 1 expression status. While there was inter‐experimental variability in numerical representation of different EV immunophenotypes distinguished by three markers used (EGFR, CD63, CD81) their patterns were reminiscent of those in panel C; (F) Immunoblotting for EGFR and EV markers in whole cell lysate and EVs of wild type GSC83 cells (WT) or their GSC83‐SYN‐KO counterparts (SYN‐KO); (G) Densitometric quantification of immunoblotting analysis of indicated EVs (*n* = 3), indicates notable diminution of ALIX and CD63 (but not EGFR, CD9 or CD81) content in EVs following disruption of syntenin 1 expression; *p* values: NS—> 0.05; *—< 0.05; **—< 0.01; ***—< 0.001; ****—< 0.0001 (see text for further details).

Syntenin 1 is considered to be a crucial element in the ESCRT‐independent biogenesis of exosomes and their related EVs (Baietti et al. 2012; Luck et al. 2020; Kugeratski et al. 2021; van Niel et al. 2022), and has been implicated in both, EGFR signalling (Du et al. 2020) and trafficking (Zanetti‐Domingues et al. 2020). To assess whether syntenin 1 is required for EV‐mediated EGFR release from glioma stem cells, GSC83 cells were engineered to disrupt SDCBP (syntenin 1) gene using CRISPR/Cas9 technology, and their EVs were assayed for EGFR content and subpopulation composition (Figure 4). Syntenin 1‐deficient cells exhibited a noticeable diminution in small EV release, as detected by NTA (Figure 4A). Interestingly, the absence of syntenin 1 altered the contributions of various tetraspanin‐expressing EV subpopulations to the GSC83 particulate secretome, as documented by the analysis of CD9‐captured EVs (ExoView; Figure 4B,C). For example, CD9‐positive EV subset co‐expressing CD63 and CD81 was reduced to 27% of the total population (from 46% ‐ Figure 2B), while EVs expressing CD81 alone expanded somewhat, as did EV subsets expressing EGFR, albeit with some variability between experiments (Figure 4B,C). A slight trend for expansion of EGFR‐positive EV subpopulations was also observed using dSTORM nanoimaging, raising from 9% (Figure 2D,E) to 12% of the total population (Figure 4D,E). It should be noted that these numbers reflect the presence of EVs with specific phenotypes, such as detectable EGFR content, and not the level of EGFR per EV, which was somewhat lower in EVs from syntenin 1‐deficient cells as suggested by bulk immunoblotting (Figure 4F,G). The composition of bulk EV‐associated proteins revealed the expected absence of Syntenin 1 and a reduction in levels of CD63 and ALIX, but little global impact on CD9, CD81 and CD44 (Figure 4F,G). Overall, these results suggest that syntenin 1‐dependent EV biogenesis pathway(s) do not critically control EGFR packaging into EVs released by GSC83 GBM stem cells.

### 3.6 Release of EGFR‐Positive EVs Depends on Neutral Sphingomyelinase Activity

**FIGURE 5:**
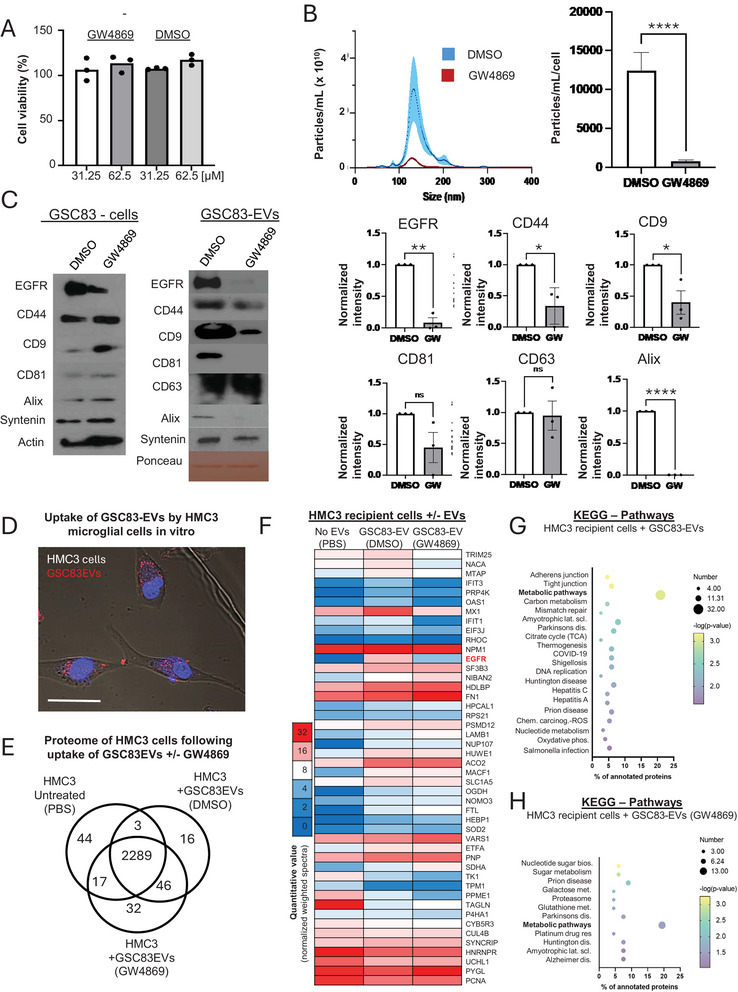
Inhibition of neutral sphingomyelinase abolishes EGFR packaging into glioma EVs, alters their molecular characteristics and functional impact on microglial recipients. (A) MTS assay documenting viability of GSC83 cells at the end of the 72‐h medium conditioning period in the presence of two different concentrations of sphingomyelinase inhibitor (GW4869) and equivalent amounts of vehicle (DMSO; *n* = 3, mean ± SD is represented). (B) NTA profiles of EV size distribution in the presence of GW4869 (50uM for 72 h) or vehicle (DMSO; left panel) and quantification of diminished EV output per GSC83 cell in the presence of GW4869 (50 µM) or DMSO following 72 h incubation (right panel: *n* = 3). (C) Western blot analysis of GSC83 cells and EVs following treatment with either GW4869 (50uM for 72 h) or vehicle documents strong depletion of EV‐associated EGFR, CD81, CD9 and ALIX (but not CD63 or syntenin 1) following treatment with the inhibitor(left panel—representative western blot; right panel—quantification; *n* = 3); (D) Confocal fluorescent microscopy documenting internalization by HMC3 human microglial cells of GSC83‐derived EVs labelled with Cell Trace CFSCE fluorescent dye (red); size bar—20 µm; (E) Venn diagram of HMC3 cellular proteins identified by mass spectrometry following treatment with EVs from either GSC83 control cells (DMSO) or from GSC83 cells pre‐treated with GW4869 (50 µM for 72 h), or cells not exposed to EVs (PBS); (F) Heatmap comparison between HMC3 cells treated with indicated subsets of GSC83 EVs or PBS documents distinct changes in recipient cell proteomes including expression of EGFR (46 proteins with highest fold change were selected from a 100 top protein subset with the greatest *p* value differential in One‐way ANOVA test); (G) KEGG pathway analysis of HMC3 protein expression following treatment with GSC83 EVs versus control (PBS); (H) KEGG pathway analysis of HMC3 protein expression following exposure to EVs from GSC83 cells pre‐treated with GW4869 or controls; *p* values: NS—> 0.05; *—< 0.05; **—< 0.01; ***—< 0.001; ****—< 0.0001 (see text for further details).

In order to further explore the known pathways of small EV biogenesis as potential contributors of the release of one or more of the EGFR‐positive EV subpopulations we treated GSC83 cells with GW4869, the well‐studied inhibitor of neutral sphingomyelinase. GW4869 compound exerts extensively documented impact on exosome biogenesis as compared to formation of membrane microvesicles (Menck et al. 2017). While GW4869 did not markedly impact growth properties of GSC83 cells (Figure 5A) it did, as expected, reduce the number of EVs detectable in culture media by NTA (Figure 5B). Interestingly, such remaining EVs were largely EGFR‐negative and were also depleted for CD44, CD9, and ALIX, but less so for CD81 and not for CD63, the latter observation being in agreement with earlier reports (Mathieu et al. 2021).

### 3.7 Distinct EV Subsets Derived From Glioma Stem Cells Exert Different Effects on Microglial Recipients

The impact of neutral sphingomyelinase inhibition on the cargo of GSC83‐derived EVs, including depletion of EGFR (Figure 5C) may suggest, that with these changes different EV subpopulations from GSC83 cells may exhibit different biological effects. To begin dissecting this question we focused on microglial cells, which account for up to 30% of the cellular mass in GBM (Quail and Joyce 2017) and represent a highly phagocytic myeloid cell lineage (Hyenne et al. 2019) with documented ability to uptake EV‐associated EGFR (Song et al. 2016). In agreement with these findings, cultured human microglial cells, HMC3, exposed to GSC83‐derived EVs pre‐labelled with fluorescent dye (CFSE) exhibited a robust accumulation of the cytoplasmatic fluorescence suggesting efficient EV internalization (Figure 5D). GSC83 cells were subsequently cultured in the presence of the vehicle (DMSO) or neutral sphingomyelinase inhibitor (GW4869), and following a three‐day exposure, their conditioned medium was collected to isolate EVs. The resulting two EV subsets included intact GSC83‐EVs and their fraction depleted for EGFR and other cargo dependent on neutral sphingomyelinase (GSC83‐EVs/GW4869). Both EV preparations were added to HMC3 cells and following three‐day culture the proteomes of untreated HMC3 cells, their counterparts treated with intact GSC83‐EVs or GSC83‐EVs/GW4869 were resolved by mass spectrometry (Figure 5E–H).

While the results of this exposure impacted only a small fraction of over 2000 detected proteins, some of the changes were intriguing. For example, intact EVs triggered EGFR expression in otherwise EGFR negative HMC3 cells, suggesting an EV‐mediated transfer of this receptor between the cells, a possibility also suggested by our earlier studies (Al‐Nedawi et al. 2008; Spinelli et al. 2024). This change was confirmed by direct WB analysis (Figure S6) but its signalling and functional consequences remain to be established (Spinelli et al. 2024). In addition, other complex molecular alterations were observed in HMC3 cells exposed to glioma EVs (Figure 5F). In this regard, more notable responses occurred among proteins involved in metabolic, RNA processing and innate cellular immunity pathways (Figure 5G).

Several of these changes were dependent on the uptake of EVs with cargo regulated by neutral sphingomyelinase, as revealed by their reversal upon GW4869 treatment of EV donor cells. For example, EGFR signal was reduced in HMC3 cells treated with GSC83‐EVs/GW4869, as expected given the drug‐sensitive content of EGFR in GSC83‐EVs (Figure 5C). The impact of GW4869 pre‐treatment on EGFR expression in EV‐treated HMC3 cells was, again, further documented by WB (Figure S6), which suggested that recipient HMC3 cells mostly acquired a glioma‐specific lower molecular weight isoform of EGFR, previously identified as EGFRvIII (Spinelli et al. 2024). This observation supports the notion that EV effects entailed an intercellular transfer of EGFR/EGFRvIII rather than induction of endogenous receptor by microglial cells. This process was sensitive to GW4869 pre‐treatment.

Other examples of the related molecular reversals associated with the influence of GW4869 on GSC83‐EVs were sought by filtering proteins detected in EV recipient (HMC3) cells for significant differences between control and drug pretreatment. Among proteins whose levels were altered in this manner, albeit to different degrees, were those associated with molecular trafficking (NACA), RNA processing (PRP4K), or innate viral immunity (TRIM25, IFIT1, IFIT3, OAS1, MX1), along with those involved in different aspects of cell metabolism (Figure 5F,H; Table S3). On the other hand, several HMC3 proteins were impacted by the exposure to GSC83‐EVs and either have undergone minimal or no change when donor cells were pre‐treated with GW4869 or became visibly up or down‐regulated as a result, likely because of the change in EV content or composition. The nature of these changes requires further clarification. Finally, we excluded the possibility that some of these changes could result from the presence of residual GW4869 compound in EV preparations by documenting that addition of excessive amounts of this drug to EV preparations does not mimic the effects of GSC83 cell pre‐treatment (Figure S6). Overall, these observations suggest that glioma cells produce multiple EV subpopulations with and without the content of driver oncoproteins, and with phenotypes and activities defined by different coexistent biogenetic and cargo loading processes.

## 4 Discussion

Our present study builds on the emerging link between oncogenic drivers involved in cancer progression and their impact on pathways/patterns of cellular vesiculation (Choi et al. 2017; Nakano et al. 2015). In this regard EGFR/EGFRvIII expressed by glioma cells often serves as a paradigm of multifaceted effects that oncogenes exert on EV biogenesis, EV‐mediated oncoprotein export (Al‐Nedawi et al. 2008), EV protein content (Choi et al. 2018), EV uptake (Lee et al. 2016), and their biological effects during cell‐cell communication (Spinelli et al. 2024). The current study focuses on the hitherto poorly studied link between EGFR/EGFRvIII oncogene expression and the scope/nature of EV heterogeneity.

In this domain we made several new observations. Thus, while GBM cells may differ with regards to the level of EGFR expression, at least in some cases, as exemplified by GSC83 cells, they are often both highly and uniformly positive for this receptor. In isolated GSC83 cells the endogenously expressed EGFR/EGFRvIII (Spinelli et al. 2024) appears to be detectable principally on the cell surface, which is evenly decorated with the EGFR antigen, as revealed by flow cytometry and confocal imaging. This expression pattern is also coupled with a strong EGFR protein signal detected in bulk EV preparations from these and several other GBM cells and their derived EVs (Al‐Nedawi et al. 2009).

While these results may suggest an abundance of EGFR‐positive EVs in the GBM cell secretome, our analysis at the single EV resolution leads to the opposite conclusion. Indeed, using three different high‐resolution instruments (nano‐flow cytometer, ExoView chip analyser, and super‐resolution nano‐imager, ONI) we document that only a minority of EVs (9%–30%) released by GSC83 cells carry EGFR on their surfaces. A somewhat greater proportion of EGFR‐positive EVs was detected in other cellular models (GSC1123 and GSC1005; Figure S1), but in all cases less than half of all EVs carried detectable amounts of this receptor. Moreover, in the case of GSC83 cells these EGFR‐positive EVs comprise at least four distinct subpopulations, as documented against their patterns of CD9, CD63 and CD81 tetraspanin expression. This may suggest that EGFR packaging into small EVs may occur along several different mechanisms, the nature of which is presently unclear.

In this context it should be mentioned that instrumental analysis of single EVs and their heterogenous populations is not free from analytical challenges. For example, nano‐flow cytometry platforms may differ considerably with respect to their limits of detection including the abundance of specific molecules, such as EGFR and tetraspanins on the surface of individual EVs, as well as thresholds of EV size detection and other factors (Kim et al. 2024). While it is possible that low levels of EGFR could be present on, what we describe as, EGFR‐negative EV subpopulations, there was a relative congruence between flow cytometry, ExoView analysis and super‐resolution microscopy, the latter operated with a molecular resolution. This comparison suggests that global profiles of EVs could be reliably extracted from all these orthogonal measurements. Moreover, our study was aimed at detection of the degree of EV heterogeneity rather than determination of the absolute presence or absence of specific markers.

It is noteworthy that the numerical frequencies of individual EVs positive or negative for specific protein markers (likely at varying levels per EV) may not predict the absolute levels of these marker proteins in the whole EV population. This is evident from comparisons between high percentages of EVs harbouring canonical tetraspanins among EGFR‐negative EVs, and the co‐purification of some of these tetraspanins (e.g., CD9) with EGFR in experiments involving magnetic beads coated with anti‐EGFRvIII antibody. While the commercial instruments employed in our study were capable of detecting distinctive EV subpopulations, thereby complementing bulk proteomics, it is very likely that with increasing dimensionality and sensitivity of the evolving single vesicle technologies a much greater depth of EV diversity could be eventually revealed and inform their analysis and applications (Rak and Strzadala 2023).

Since internalization and intracellular trafficking of EGFR depends on the intensity of its activation or the existence of activating mutations (Tomas et al. 2014), it is possible that different EV subsets may contain different levels and isoforms of EGFR, a question which remains unanswered and deserves further study. The use of calibrated levels of fluorophores, antigen quantification, and the usage of optimized conjugated antibodies, or nanobodies, would potentially enable a greater precision in detection of EGFR levels on individual EVs (Welsh et al. 2020). While these important questions await a detailed analysis our results consistently point to a puzzling scarcity of EGFR‐high EVs in the secretome of uniformly EGFR‐high cancer cells.

This observation has several noteworthy implications. First, the relative absence of EGFR on the surface of (some) cancer‐derived EVs does not preclude the expression of EGFR (or other marker molecules) by the cancer cells themselves. This is of considerable relevance in the context of liquid biopsy applications in GBM and in other settings (Indira Chandran et al. 2024), which may require investment in multiplexing to define or capture tumour derived EVs (Rak and Strzadala 2023) beyond what is considered a crucial cancer‐specific marker, such as EGFR/EGFRvIII. Second, the existence of EGFR‐negative EVs in the secretome of EGFR/EGFRvIII‐driven cancer cells raises questions as to their biological roles and interactions (competition or cooperation) between different EV subpopulations, as alluded to in the literature (Adnani et al. 2022). Similarly, whether different EGFR‐containing EV subpopulations contribute to the same or different biological effects ascribed to EV‐associated EGFR (Al‐Nedawi et al. 2008; Spinelli et al. 2024) remains an interesting and presently unresolved question.

In this regard our analysis involving intact or GW4869‐modified EV subpopulations suggests different biological responses in recipient cells. For example, the protein repertoire of microglial HMC3 cells revealed marked differences in response to intact glioma EVs versus those that were depleted for neutral sphingomyelinase‐sensitive cargo, including EGFR. While protein labelling may be required to discern which elements of the HMC3 proteome were induced, and which were transferred with GSC83‐EV (Li et al. 2023), the latter is likely the case for EGFR, which is not readily detectable in EV‐untreated HMC3 proteome. This protein was only scarcely detected in HMC3 recipient cells if they were exposed to EGFR‐negative EVs from GW4869‐treated donors. It is intriguing that even a relatively small fraction of EGFR‐positive EVs can mediate an effective transfer of this oncogenic receptor to microglial recipients. The nature of glioma EV effects on microglial cells is also of great interest as the changes we observed were not consistent with inflammatory activation, or proliferation of these myeloid cells abundant in the GBM microenvironment (Quail and Joyce 2017). Rather, these responses resembled metabolic adjustments (Bernier et al. 2020) with intriguing contribution of pathways involved RNA processing and innate pathogen responses. Understanding the biological consequences of these changes requires further study.

It should also be noted that EV preparations generated in the course of our experiments, could contain admixtures of non‐vesicular particles (NVEP), co‐purified with EVs due to the nature of our EV isolation protocol (Jeppesen et al. 2019). This notion is of special interest in the case of EGFRvIII‐negative particulate fraction obtained following incubation of EV isolates with anti‐EGFRvIII‐coated magnetic beads. The enrichment of this fraction in proteasome proteins and histones and relatively low levels of tetraspanins (CD9), ESCRT‐related proteins (TSG101), and membrane proteins (CD44) may suggest that this EGFRvIII‐depleted material may contain distinctive particulates hitherto poorly characterized. In this regard, Kim et al. have recently described EVs carrying elements of the proteasome and capable of transferring them to recipient cells with functional consequences (Kim et al. 2025). Similarly, EVs loaded with nuclear content (Lee et al. 2014; Kahlert et al. 2014; Thakur et al. 2014), including histones have been previously reported (Wortzel et al. 2024; Singh et al. 2025). Moreover, it is likely that EGFRvIII‐depleted particulates could also include bone fide NVEPs (e.g., exomeres or supermeres), although we have not used centrifugation forces usually required for enrichment of these small structures (Zhang et al. 2021) and electron microscopy revealed EV‐like structures in these preparations (Figure 3B). Many of these questions require further, more extensive studies in the future, including high resolution density gradient fractionation, super resolution microscopy and biochemical analyses.

While this aspect would not affect our analysis of EGFR or EV heterogeneity, such accompanying NVEPs could exert some impact on the responses of HMC3 cells treated with particulates derived from glioma cells. This question deserves special consideration in the context of GW4869 treatment, which in our hands strongly suppressed the numbers of EVs produced by GSC83 cells. Such conditions could change the balance between EVs dependent on neutral sphingomyelinase and other particles, including NVEPs. However, our NTA, imaging and marker analysis data suggest that the predominant fraction of our isolates contained objects with properties consistent with those of small EVs, including size distribution, morphology and strong presence of membrane molecules, including tetraspanins. The latter was especially notable in EV pellets following treatment with GW4869. It is certainly of great future interest to dissect the responses of microglial cells to different subsets of tumour‐derived vesicles and particles and their natural combinations.

It is noteworthy that, in spite of the apparent diversity among EGFR‐positive EV subsets they all depended on neutral sphingomyelinase, but were largely unaffected by the removal of syntenin 1, both linked to ESCRT‐independent mechanisms of exosome biogenesis (Chi et al. 2020). A complete and selective removal of EGFR‐positive EVs from the particulate secretome of GSC83 cells using immune‐depletion protocol revealed a subset of EVs/particles enriched for proteasome proteins and histones, suggesting, again, the involvement of different cargo packaging mechanisms and possibly different subcellular region from which such EGFR‐negative EVs/particles might originate.

EGFR can be exported as cargo of EVs in several ways including endocytosis followed by exosome formation (Zanetti‐Domingues et al. 2020), incorporation into larger microvesicles released from the plasma membrane (Al‐Nedawi et al. 2008; Di Vizio et al. 2009) and through generation of large and small apoptotic vesicles (Montermini et al. 2015; Choi et al. 2019). The intracellular processing of EGFR may also depend on the mode of receptor activation (Tomas et al. 2014) and is likely affected by the cell type and related signalling peculiarities, including cases where, as in GBM (especially in GSC83 cells) wild type EGFR is co‐expressed with ample amounts of EGFRvIII, resulting in molecular interactions potentially impacting receptor internalization and EV biogenesis (An et al. 2018).

Our initial observations may suggest that in the context of EGFR/EGFRvIII‐driven mesenchymal glioma stem cells several mechanisms of EV biogenesis may operate simultaneously albeit with some common molecular nodes. This is for several reasons. First, EGFR was associated with several different repertoires of small EV‐ and exosome‐related tetraspanins (CD9, CD63, CD81) (Kowal et al. 2016), including EVs in which none of them were detectable. Second, targeting neutral sphingomyelinase (GW4869), which is often considered to be a part of the ESCRT‐independent EV biogenetic machinery (van Niel et al. 2018; Bianco et al. 2009) depleted the EGFR‐positive EV subpopulations indicating a possibility of this pathway being involved in EGFR loading. Third, syntenin 1‐deficient glioma cells, which would be expected to exhibit impaired ESCRT‐independent exosome biogenesis (Baietti et al. 2012; van Niel et al. 2018), continued to produce small, EGFR‐positive EVs, as well as their EGFR‐negative counterparts.

Disruption of syntenin 1 did not eliminate any of the pre‐existing EV subpopulations harbouring distinct repertoires of tetraspanins, but it altered somewhat their contributions to the particulate secretome of cancer cells. Of note, syntenin 1‐deficient cells released reduced proportion of EVs double positive for CD63 and CD81, as revealed by ExoView (CD9 capture) analysis and by super‐resolution microscopy. This is consistent with the postulated role of the PDZ domain of syntenin 1 as the site of interaction with C‐terminal domain of CD63 (Latysheva et al. 2006) and the ability of CD63 to then pull CD81 tetraspanin into the syntenin 1‐dependent EV biogenesis pathway (Ai et al. 2024). However, other studies suggest that syntenin 1‐dependent exosome biogenesis (Baietti et al. 2012) may involve CD63, but not CD81 or CD9 tetraspanins (Roucourt et al. 2015). Syntenin 1 was also recently reported to drive formation of CD63‐positive EVs at the cellular plasma membrane (Ai et al. 2024), or serve as a protein hub for multiple components of vesiculation (Luck et al. 2020; Kugeratski et al. 2021). In this context it is puzzling that the relative number of CD63‐only EVs was visibly elevated (and not diminished) in the secretome of syntenin 1 deficient cells. A slight increase was also observed in the proportion of CD81‐only and EGFR‐containing EVs amidst somewhat reduced overall EV output. Since this shift was not evident among global levels of the respective EV proteins measured in bulk (Western blotting), it could be suggested that changes at the single EV level (ExoView) unmask the realignment of EV biogenesis pathways once a specific element of this circuitry has been suppressed. Further studies with additional markers and higher dimensionality of single EV analysis could shed more light on the branching of EV biogenesis pathways, including the nature, subcellular origin, formation and biological activity of distinct EV subpopulations released by specific cancer cells.

While our study focuses on the distribution of marker proteins across different EV subpopulations addition of RNA and other biomolecules could further increase the granularity of EV landscapes (Spinelli et al. 2024). However, the low copy number of specific RNA biotypes per EV pose considerable challenges (Chevillet et al. 2014) in this regard, as do complexities of techniques enabling RNA analysis at the single EV level (de Voogt et al. 2021; Oliveira et al. 2020).

Overall, our study suggests that bulk EV proteomics (Greening et al. 2016; Hoshino et al. 2020), while often highly informative, may obscure the important complexities of EV subpopulations (Rak and Strzadala 2023), each of which may possess unique features, diagnostic utility and biological activity in cancer and beyond. Studies involving the emerging single EV proteomics or transcriptomics, especially with higher dimensionality (Shao et al. 2018; Jalali et al. 2023), are likely to reveal collective features of complex EV ‘landscapes’ (Rak and Strzadala 2023) that ultimately shape their diagnostic utility and functional properties.

## Author Contributions

**Elham Pishavar**: conceptualization, methodology, validation, formal analysis, investigation, data curation, visualization, writing – original draft. **Fereshteh Shojaei‐Ghahrizjani**: conceptualization, methodology, formal analysis, investigation, data curation. **Brian Meehan**: conceptualization, methodology, data curation, investigation, writing – original draft, visualization, supervision. **Nadim Tawil**: conceptualization, methodology, validation, formal analysis, investigation, data curation, visualization, writing – original draft, supervision. **Cristiana Spinelli**: conceptualization, methodology, resources, writing – original draft. **Dongsic Choi**: methodology, validation, formal analysis, data curation, visualization, writing – original draft. **Fang Cheng Wong**: methodology, validation, investigation, data curation, visualization, writing – original draft. **Mahsa Jalali**: conceptualization, methodology, resources, validation, formal analysis, investigation, data curation, visualization, writing – original draft. **Janusz Rak**: conceptualization, supervision, data curation, visualization, writing – original draft, writing – review and editing, supervision, project administration, funding acquisition.

## Ethics Statement

This study adheres to all institutional and international research ethics guidelines. No animal or human subject research is included in this work.

## Conflicts of Interest

The authors declare no conflicts of interest.

## Supporting information




**Supplementary Figure 1**. Heterogeneity of single extracellular vesicle (EV) subpopulations as measured by the ExoView platform. EVs from two different mesenchymal glioma stem cell lines (GSCs) were captured on anti‐CD9 antibody and probed with antibodies against CD63, CD81 and EGFR. This staining revealed the existence of seven different EV phenotypes with different representation in the total population as indicated by numbers in/under the corresponding circle plots. A. EVs from GSC1123 cells; B. EVs from GSC1005 cells (see text and Figures 2 and 3 for details).
**Supplementary Figure 2**. Gene Ontology (GO) enrichment analysis of differentially expressed proteins in EGFRvIII‐positive GSC83‐derived EV samples. (A) Mass spectrometry data were subjected to Gene Ontology biological process (BP) enrichment analysis of proteins significantly upregulated in samples of EGFR‐positive EVs captured on anti‐EGFR‐coated beads. (B) Gene Ontology molecular function (MF) enrichment analysis of proteins significantly upregulated in EGFR‐positive samples.
**Supplementary Figure 3**. Gene Ontology (GO) enrichment analysis of differentially expressed proteins in EGFRvIII‐negative GSC83‐derived EV samples. (A) Mass spectrometry data were subjected to Gene Ontology biological process (BP) enrichment analysis of proteins significantly upregulated in EVs not captured by anti‐EGFR coated beads (EGFR‐negative samples). (B) Gene Ontology molecular function (MF) enrichment analysis of proteins significantly upregulated in EGFR‐negative samples.
**Supplementary Figure 4**. KEGG pathway enrichment analysis of EGFRvIII‐positive GSC83‐Derived EVs (mass spectrometry). Bubble plots represent significantly enriched pathways identified by Kyoto Encyclopedia of Genes and Genomes (KEGG) pathway enrichment analysis in EVs captured by anti‐EGFRvIII‐coated magnetic beads (EGFR‐positive). Each bubble corresponds to an enriched pathway. Bubble size reflects the number of proteins/genes mapped to each pathway (count), and colour intensity represents the statistical significance (‐log10 adjusted *p*‐value). Only pathways meeting the significance threshold (adjusted p < 0.05) are shown.
**Supplementary Figure 5**. KEGG pathway enrichment analysis of EGFR‐negative GSC83‐Derived EVs (mass spectrometry). Bubble plots represent significantly enriched pathways identified by Kyoto Encyclopedia of Genes and Genomes (KEGG) pathway enrichment analysis in EVs not captured by anti‐EGFRvIII‐coated beads. Each bubble corresponds to an enriched pathway. Bubble size reflects the number of proteins/genes mapped to each pathway (count), and colour intensity represents the statistical significance (‐log10 adjusted *p*‐value). Only pathways meeting the significance threshold (adjusted p < 0.05) are shown.
**Supplementary Figure 6**. The levels of EGFR expression in HMC3 microglial cells exposed to extracellular vesicles (EVs) from glioma stem cells pre‐treated with GW4869. This immunoblot represents a validation of mass spectrometry data depicted in Figure 4, indicating changes in the effects of EVs on recipient cell proteome, including a diminished transfer of EGFR, when EV donor cells were pre‐treated with GW4869. Protein was extracted from HMC3 cells that were incubated with either PBS alone (lane 1) or with EVs from GSC83 cells exposed to vehicle (DMSO; lane 2) or GW4869 (50 µM; lane 5) prior to EV collection. HMC3 cells were also incubated with control GSC83‐EVs (DMSO) mixed with GW4869 at the indicated concentrations (20 and 50 µM). Equal loading was visualized by probing for beta‐actin and Ponceau staining (bottom panel). Representative data are shown from three independent repeats.

**Supplementary Table 1**. Proteome of glioma extracellular vesicles enriched or depleted for EGFR—significant differences

**Supplementary Table 2**. Proteome of glioma extracellular vesicles enriched or depleted for EGFR—all proteins

**Supplementary Table 3**. Proteome of microglial HMC3 cells following treatment with glioma stem cell derived extracellular vesicles (EVs).

## Data Availability

The data that support the findings of this study will be openly available on request
