# Supplementary material for: Glioblastoma Cells Expressing Oncogenic EGFR Release Multiple Extracellular Vesicle Subpopulations Positive or Negative for EGFR
Source: J Extracell Biol. 2026 Apr 7;5(4):e70134. doi: 10.1002/jex2.70134 (PMC13054837; doi:10.1002/jex2.70134)
Supplement: Supplementary file 1 — Supplementary Figure 1. Heterogeneity of single extracellular vesicle (EV) subpopulations as measured by the ExoView platform. EVs from two different mesenchymal glioma stem cell lines (GSCs) were captured on anti‐CD9 antibody and probed with antibodies against CD63, CD81 and EGFR. This staining revealed the existence of seven different EV phenotypes with different representation in the total population as indicated by numbers in/under the corresponding circle plots. A. EVs from GSC1123 cells; B. EVs from GSC1005 cells (see text and Figures 2 and 3 for details). Supplementary Figure 2. Gene Ontology (GO) enrichment analysis of differentially expressed proteins in EGFRvIII‐positive GSC83‐derived EV samples. (A) Mass spectrometry data were subjected to Gene Ontology biological process (BP) enrichment analysis of proteins significantly upregulated in samples of EGFR‐positive EVs captured on anti‐EGFR‐coated beads. (B) Gene Ontology molecular function (MF) enrichment analysis of proteins significantly upregulated in EGFR‐positive samples. Supplementary Figure 3. Gene Ontology (GO) enrichment analysis of differentially expressed proteins in EGFRvIII‐negative GSC83‐derived EV samples. (A) Mass spectrometry data were subjected to Gene Ontology biological process (BP) enrichment analysis of proteins significantly upregulated in EVs not captured by anti‐EGFR coated beads (EGFR‐negative samples). (B) Gene Ontology molecular function (MF) enrichment analysis of proteins significantly upregulated in EGFR‐negative samples. Supplementary Figure 4. KEGG pathway enrichment analysis of EGFRvIII‐positive GSC83‐Derived EVs (mass spectrometry). Bubble plots represent significantly enriched pathways identified by Kyoto Encyclopedia of Genes and Genomes (KEGG) pathway enrichment analysis in EVs captured by anti‐EGFRvIII‐coated magnetic beads (EGFR‐positive). Each bubble corresponds to an enriched pathway. Bubble size reflects the number of proteins/genes mapped to each pathway (count), a [file JEX2-5-e70134-s003.pdf]

A

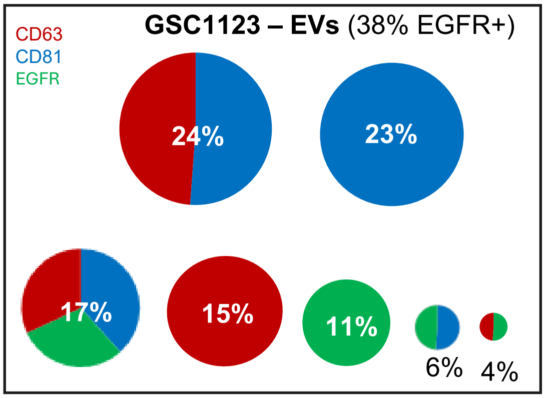

B

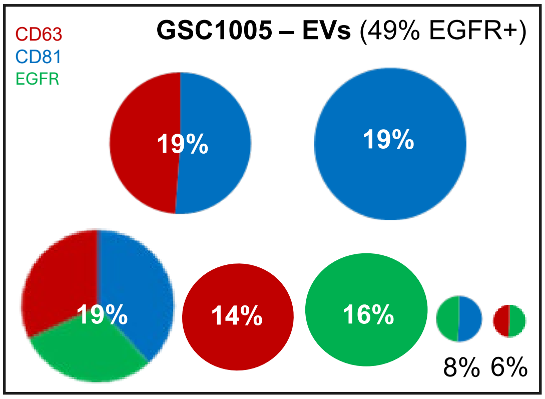

Supplementary Figure 1. Heterogeneity of single extracellular vesicle (EV) subpopulations as measured by the ExoView R200 platform. EVs from two different mesenchymal glioma stem cell lines (GSCs) were captured on anti-CD9 antibody and probed with antibodies against CD63, CD81 and EGFR. This staining revealed the existence of six different EV phenotypes with different representation in the total population as indicated by numbers in/under the corresponding circle plots. (**A**) EVs from GSC1123 cells; (**B**) EVs from GSC1005 cells (see text and Figures 2 and 3 for details).

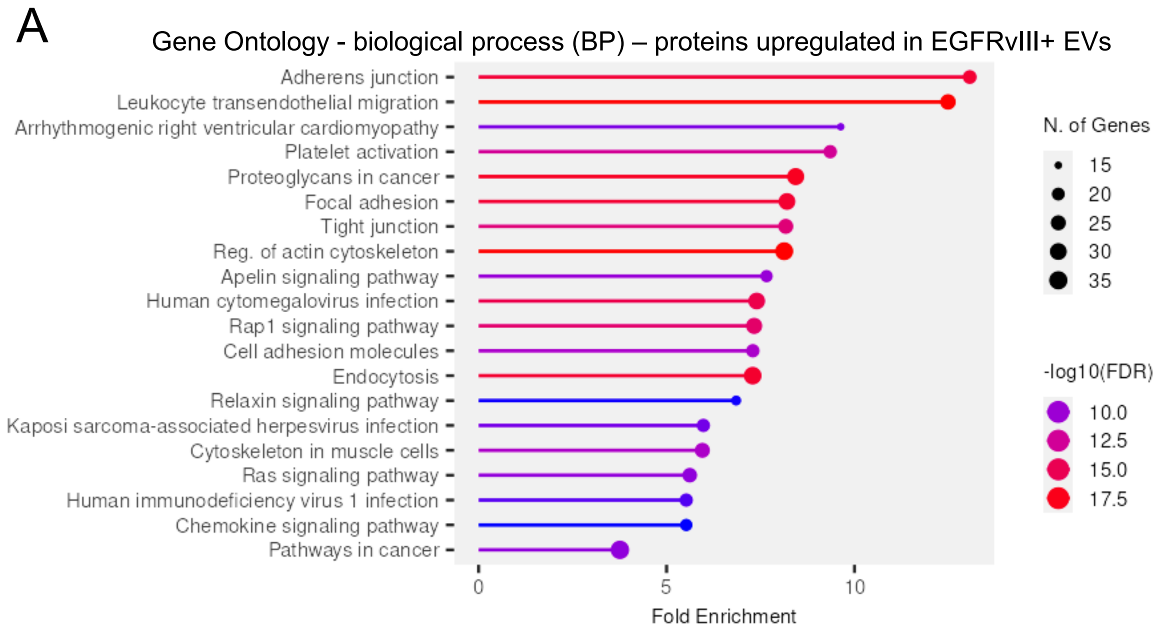

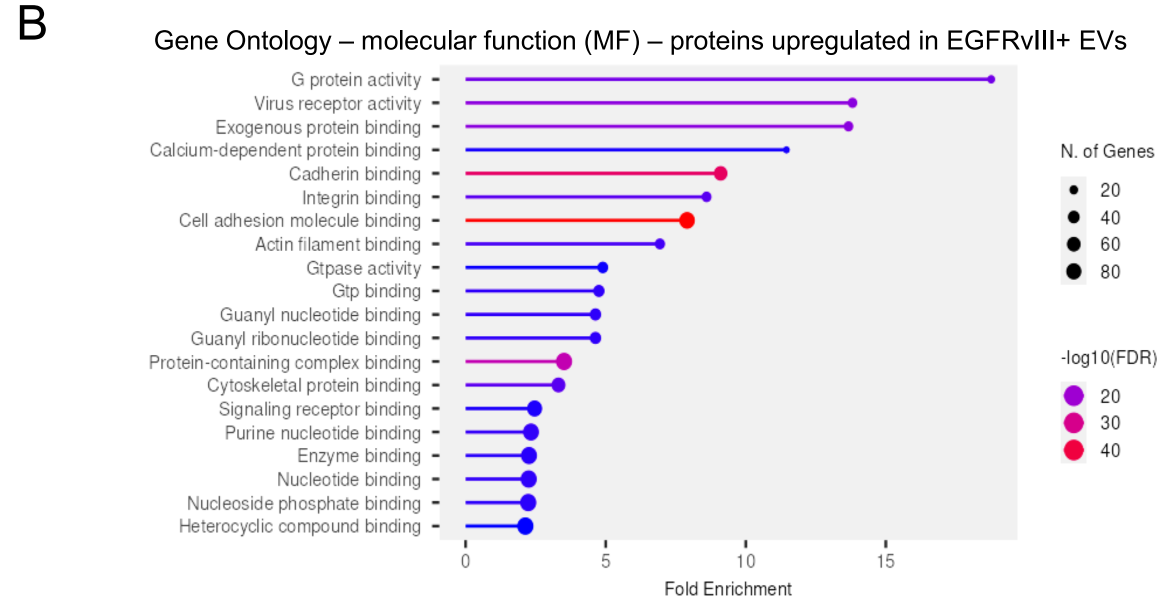

Supplementary Figure 2. Gene Ontology (GO) enrichment analysis of differentially expressed proteins in EGFRvIII-positive GSC83-derived EV samples. (A) Mass spectrometry data were subjected to Gene Ontology biological process (BP) enrichment analysis of proteins significantly upregulated in samples of EGFR-positive EVs captured on anti-EGFR-coated beads. **(B)** Gene Ontology molecular function (MF) enrichment analysis of proteins significantly upregulated in EGFR-positive samples.

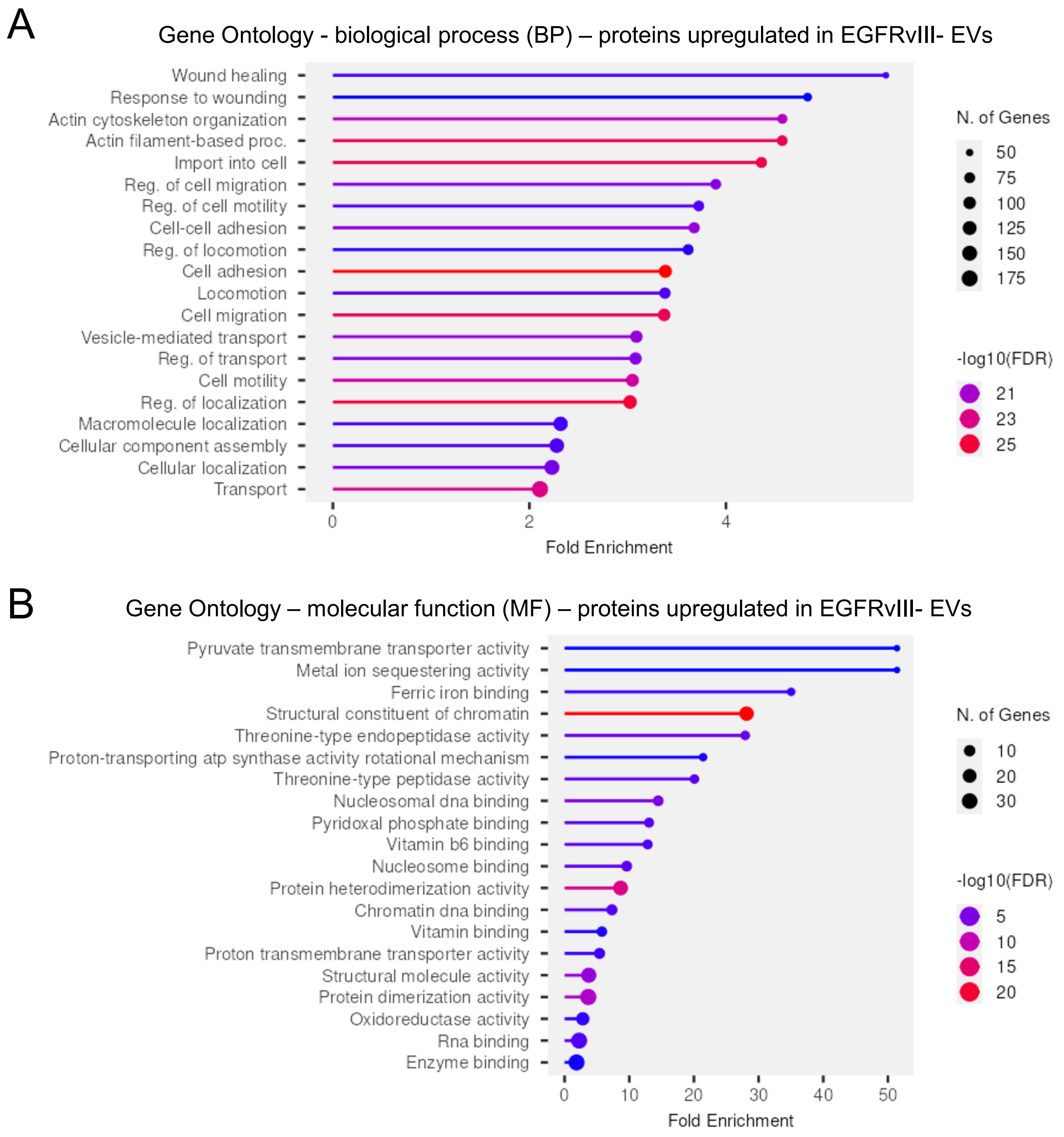

Supplementary Figure 3. Gene Ontology (GO) enrichment analysis of differentially expressed proteins in EGFRvIII-negative GSC83-derived EV samples. (A) Mass spectrometry data were subjected to Gene Ontology biological process (BP) enrichment analysis of proteins significantly upregulated in EVs not captured by anti-EGFR coated beads (EGFR-negative samples). (**B)** Gene Ontology molecular function (MF) enrichment analysis of proteins significantly upregulated in EGFR-negative samples.

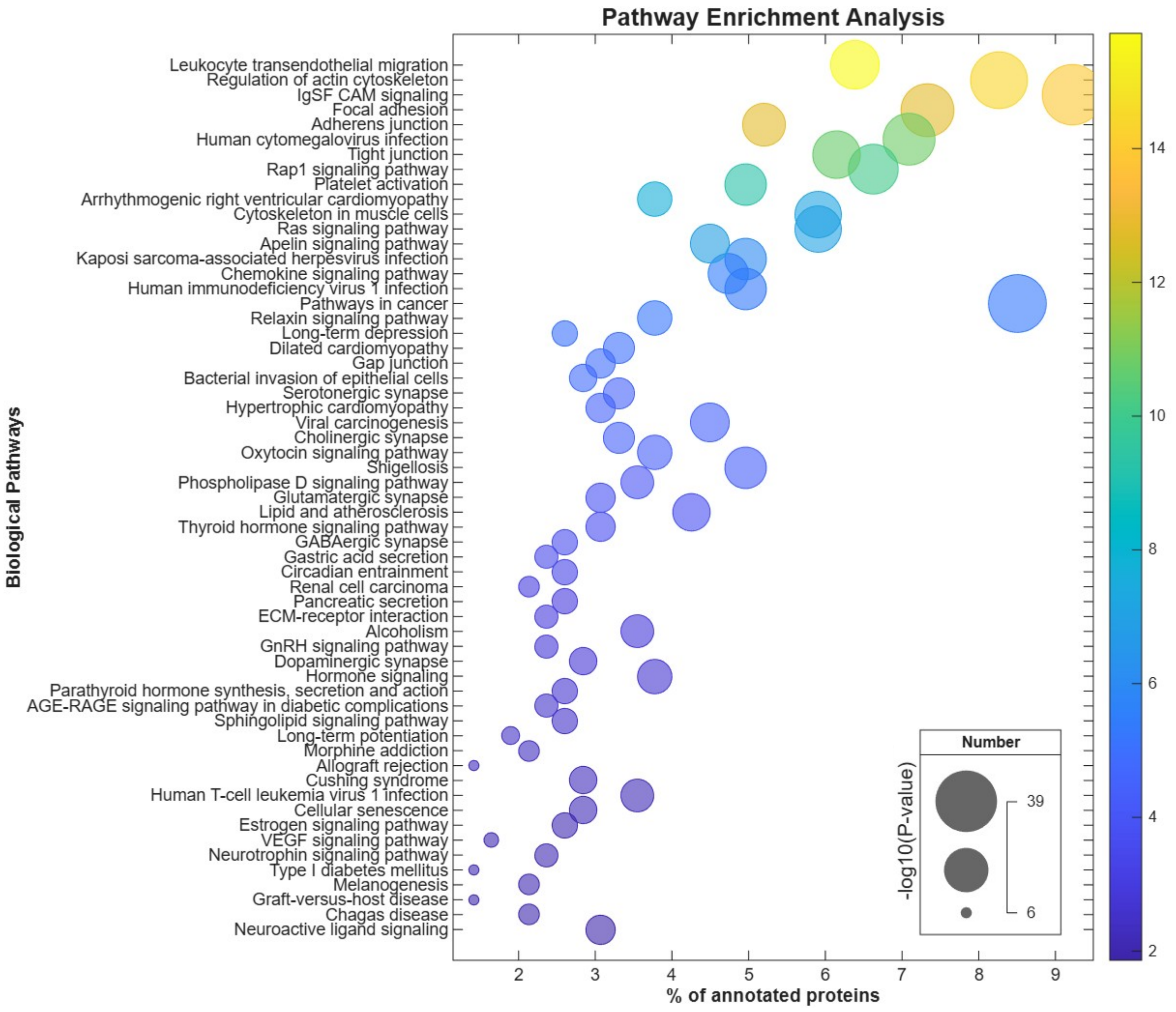

Supplementary Figure 4. KEGG pathway enrichment analysis of EGFRvIII-positive GSC83-Derived EVs (mass specgtrometry). Bubble plots represent significantly enriched pathways identified by Kyoto Encyclopedia of Genes and Genomes (KEGG) pathway enrichment analysis in EVs captured by anti-EGFRvIII-coated magnetic beads (EGFR-positive). Each bubble corresponds to an enriched pathway. Bubble size reflects the number of proteins/genes mapped to each pathway (count), and colour intensity represents the statistical significance (-log10 adjusted p-value). Only pathways meeting the significance threshold (adjusted $p < 0.05$) are shown.

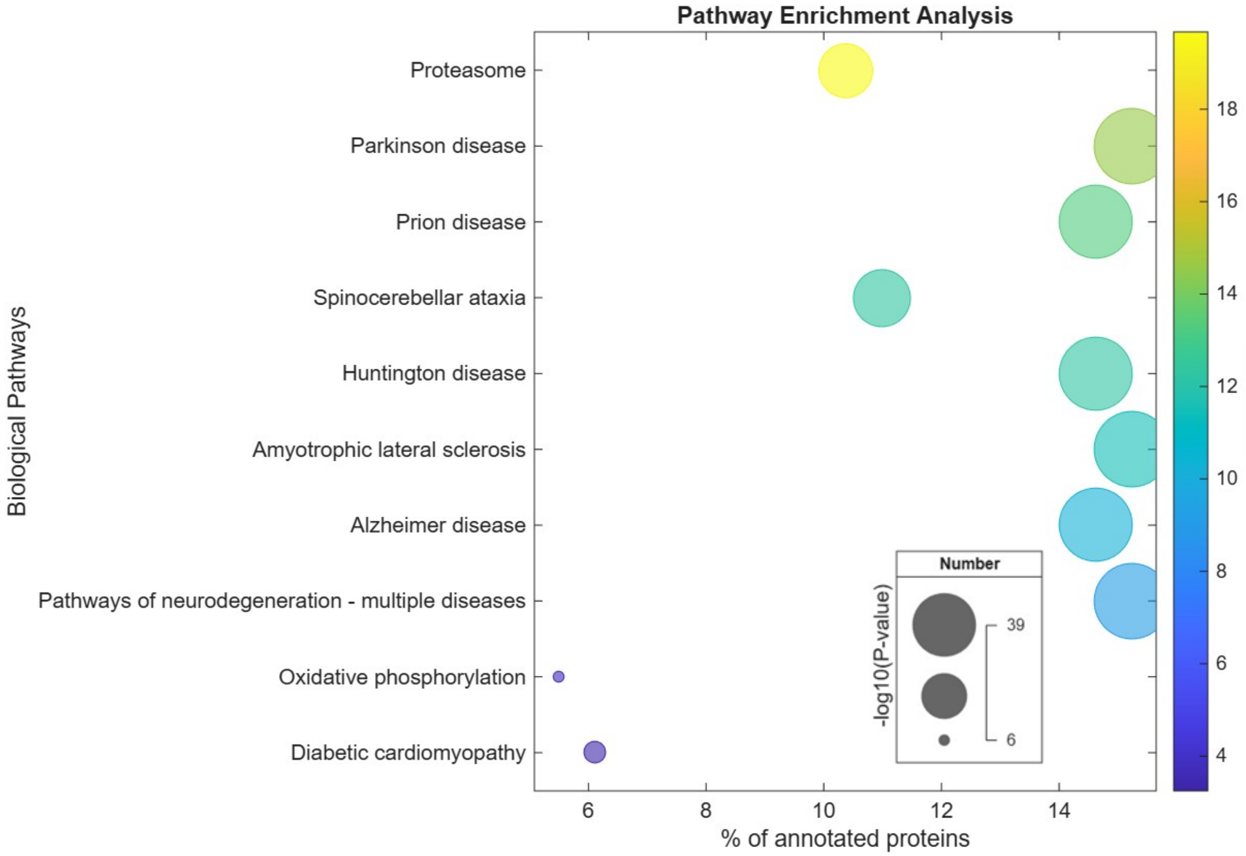

Supplementary Figure 5. KEGG pathway enrichment analysis of EGFR-negative GSC83-Derived EVs (mass spectrometry). Bubble plots represent significantly enriched pathways identified by Kyoto Encyclopedia of Genes and Genomes (KEGG) pathway enrichment analysis in EVs not captured by anti-EGFRvIII-coated beads. Each bubble corresponds to an enriched pathway. Bubble size reflects the number of proteins/genes mapped to each pathway (count), and color intensity represents the statistical significance (-log10 adjusted p-value). Only pathways meeting the significance threshold (adjusted $p < 0.05$) are shown.

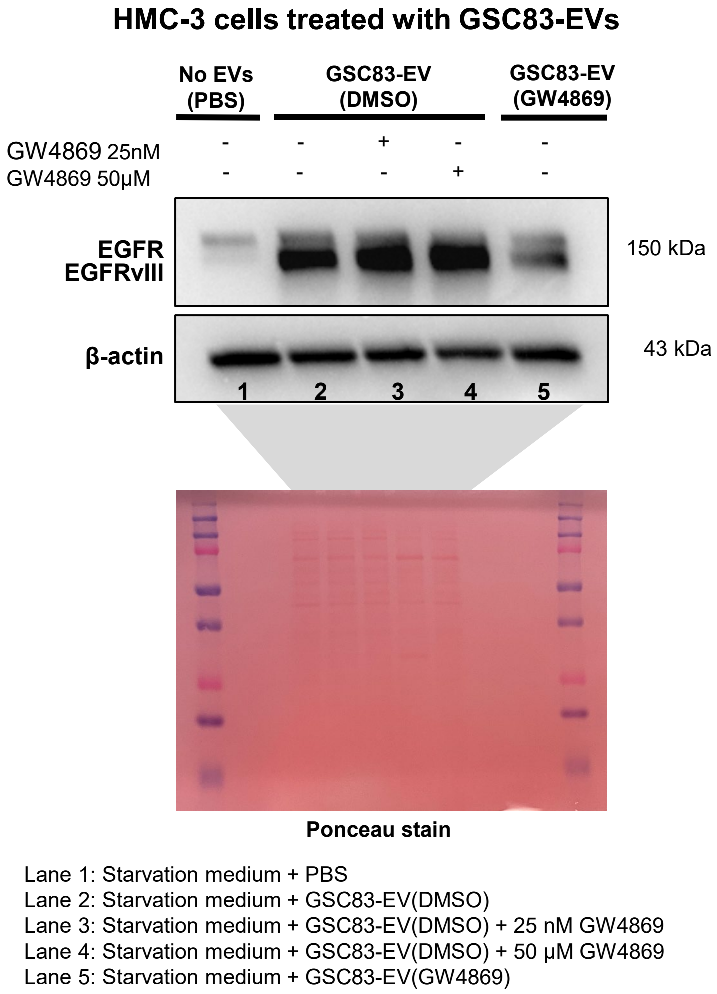

Supplementary Figure 6. The levels of EGFR expression in HMC-3 microglial cells exposed to extracellular vesicles (EVs) from glioma stem cells pre-treated with GW4869. This immunoblot represents a validation of mass spectrometry data depicted in Figure 4, indicating changes in the effects of EVs on recipient cell proteome, including a diminished transfer of EGFR, when EV donor cells were pre-treated with GW4869. Protein was extracted from HMC-3 cells that were incubated with either PBS alone (lane 1) or with EVs from GSC83 cells exposed to vehicle (DMSO; lane 2) or GW4869 (50 µM; lane 5) prior to EV collection. HMC-3 cells were also incubated with control GSC83-EVs (DMSO) mixed with GW4869 at the indicated concentrations (20µM and 50 µM). Representative data are shown from three independent repeats.
